# Truthmaker Semantics for Intuitionistic Modal Logic

**DOI:** 10.1007/s11245-024-10094-z

**Published:** 2024-11-29

**Authors:** Jon Erling Litland

**Affiliations:** 1 Department of Philosophy, University of Texas at Austin, 2210 Speedway, WAG 316, Stop C3500, Austin, TX 78712 USA; 2 IFIKK, University of Oslo, Postboks 1020 Blindern 0315, Oslo, Norway

**Keywords:** Truthmaker Semantics, Modality, Intuitionistic Logic, Intuitionistic Modal Logic

## Abstract

A truthmaker for a proposition *P* is *exact* if it contains nothing irrelevant to *P*. What are the exact truthmakers for necessitated propositions? This paper makes progress on this issue by showing how to extend Fine’s truthmaker semantics for intuitionistic logic to an exact truthmaker semantics for intuitionistic modal logic. The project is of interest also to the classical logician: while all distinctively classical theorems may be true, they differ from the intuitionistic ones in how they are made true. This sheds new light on the status of the **T** and **B** axioms.

## Introduction

If it is necessary that Socrates is human, what *makes* it necessary that Socrates is human? More generally, if a proposition *P* is necessarily true, what makes it the case that *P* is necessarily true? In short, what are the *truthmakers* for necessitated propositions?^1^ Philosophers have put forward a number of metaphysically substantive answers to these questions.^2^ Our goal in this paper is in one way more modest: we will not propound a substantive view about the truthmakers for necessitated propositions, rather we will just develop an exact truthmaker semantics for the necessity operator $\Box$.^3^ In another way our goal is more ambitious: the truthmaker semantics should be adequate not just when $\Box$ is interpreted as metaphysical necessity, but also when it is interpreted as, say, nomological necessity, deontic necessity, or knowledge.

Throughout the main text the presentation is kept at an informal level; for the full technical details the reader is referred to appendix A. For the reader’s benefit, here’s an overview of the main paper. We begin in §2 by rehearsing the basics of the truthmaker semantics for intuitionistic propositional logic (Fine 2014), highlighting the idea that while all the theorems of classical logic might be *true*, they require substantial truthmakers; in contrast, the theorems of intuitionistic logic are all made true by the null truthmaker. A classical logician may prefer the more standard bilateral treatment of negation; §2.1 briefly considers what the distinction between substantial and insubstantial truths look like in a such a setting. Turning next to truthmakers for modal propositions, in §3 we show how to construct a modal truthmaker semantics based on ideas from neighborhood semantics. It turns out that all theorems of the intuitionistic modal logic $\textbf{IntK}_{\Box }$—the analogue of the smallest classical normal modal logic—have the null truthmaker and are in that sense insubstantial. In §4 we consider whether we can achieve the same result using *accessibility relations*. One of the main philosophical contributions of this paper is an argument (§§4.1 to 4.3 that the possibility of necessary connections between distinct propositions rules this out. In §5 we consider extensions of $\textbf{IntK}_{\Box }$. The second important philosophical contribution of the paper is an account of how different modalities might be characterized by the same principles, but differ in how those principles are made true. The main paper ends in §6 by discussing some avenues for further research. Appendix A puts the claims in the main paper on a rigorous footing and establishes soundness and completeness results.

## Truthmaker Semantics for Intuitionistic Logic

In possible worlds semantics propositions are simply true (false) at a world; truthmaker semantics looks inside the world to find the *states* that *make* propositions true (or false). Unlike worlds, states are, in general, neither *complete* nor *consistent*: they may fail to make true both a proposition and its negation and they may make true both a proposition and its negation.

Unlike worlds, states also stand in interesting mereological relationships. We write $s \sqsubseteq t$ to mean that the state *s* is part of the state *t*. For any collection $S_0$ of states there is a smallest state that has each member of $S_0$ as a part; this is the *fusion* of the states $S_0$. We write $\bigsqcup S_0$ for the fusion of $S_0$; if $S_{0}= \left\{ s_0, \dotsc , s_{n-1}\right\}$ is finite we write $s_{0 }\sqcup s_1 \cdots \sqcup s_{n-1}$ for $\bigsqcup \left\{ s_0, \dotsc , s_{n-1}\right\}$. Note that $\bigsqcup \emptyset$ exists; this is the *null state*, the minimal truthmaker. Henceforth we write “$0$” for this state—it will play an important role in what follows.

Throughout we are interested in *exact* truthmaking.^4^ Informally, if *s* makes *P* true, then every part of *s* is relevant to making *P* true. The mathematical cash value is that containing a truthmaker for a proposition does not entail being a truthmaker for that proposition. If *t* has a part that is an exact truthmaker for *P*, say that *t* is an *inexact* truthmaker for *P*.

The goal of truthmaker semantics is to specify the truthmakers for complex propositions in terms of the truthmakers for less complex ones. For conjunction and disjunction it is clear what to say. A truthmaker for a conjunction $P \wedge Q$ is a fusion of a truthmaker for *P* with a truthmaker for *Q*; a truthmaker for a disjunction $P \vee Q$ is either a truthmaker for *P* or a truthmaker for *Q*.^5^ But negation poses notorious problems: how can one determine the truthmakers for a negation $\lnot P$ on the basis of the truthmakers for *P*? In this paper we follow Fine 2014 in adopting an intuitionistic treatment of negation. We define $\lnot P$ as $P \rightarrow \bot$; here $\bot$ is some designated absurd proposition and $\rightarrow$ is an intuitionistic conditional.

Say that *P* *inexactly entails* *Q* iff every truthmaker for *P* contains a truthmaker for *Q*. Following Fine we take there to be a collection *C* of contradictory states, which we take to be the truthmakers for $\bot$. A state that contains a contradictory state is said to be *inconsistent*. Since the intuitionist accepts the rule of *ex falso quodlibet* we must ensure that $\bot$ inexactly entails each proposition. To do this we follow Fine and define propositions to be sets of states *P* such that every contradictory state contains an element of *P*.

The intuitionistic conditional is an “incremental” conditional in the sense of Yablo 2018; 2016 and Fine 2020. A truthmaker for $P \rightarrow Q$ is a state *s* such that *s* contains exactly the “increments” that you have to “add” to a truthmaker for *P* in order to obtain a truthmaker for *Q*. Following Fine 2014 we implement this by assuming that for any two states *s*, *t* there is a smallest state $s \dashrightarrow t$ such that the fusion of $s \dashrightarrow t$ with *s* contains *t*. This is the “conditional connection” between *s* and *t*. (See further appendix A.3 and appendix A.4.) Let *f* be a function from truthmakers for *P* to truthmakers for *Q*. The truthmakers for $P \rightarrow Q$ are then of the form $\bigsqcup _{s \text { a verifier for P}} s \dashrightarrow f(s)$.

Consider a propositional language $\mathcal {L}$ with connectives $\wedge , \vee , \rightarrow , \bot$. A *truthmaker-interpretation* of $\mathcal {L}$ consists in a set *S*, a parthood relation $\sqsubseteq$ on *S*, a subset $C \subseteq S$ of contradictory states, and an interpretation function ${\llbracket \phantom {x} \rrbracket }$ assigning a proposition to each sentence such that ${\llbracket \bot \rrbracket } =C$; ${\llbracket \phi \wedge \psi \rrbracket } = {\llbracket \phi \rrbracket } \wedge {\llbracket \psi \rrbracket }$; ${\llbracket \phi \vee \psi \rrbracket } = {\llbracket \phi \rrbracket } \vee {\llbracket \psi \rrbracket }$; and ${\llbracket \phi \rightarrow \psi \rrbracket } = {\llbracket \phi \rrbracket } \rightarrow {\llbracket \psi \rrbracket }$. Fine 2014 establishes the following two philosophically suggestive results about such interpretations (see Theorem A.2 below).

First, he established that the theorems of intuitionistic logic are *null-valid*. That is, if $\phi$ is a theorem of intuitionistic logic, then $\phi$ is always interpreted as a proposition that is verified by the null state. This is metaphysically suggestive. Think of a proposition *P* as making a demand on reality: reality has to contain a collection of states that fuse to a truthmaker for *P*. Given that the null state is the fusion of the empty collection of propositions, a proposition that is verified by the null state thus makes the empty demand. Fine’s result thus suggests that the truths of intuitionistic logic are insubstantial, in that they make the empty demand on how reality is.

Fine’s second result concerns classical logic. An instance $\phi \vee \lnot \phi$ of excluded middle makes a substantive demand on reality: it has to contain either a truthmaker for $\phi$ or a truthmaker for $\lnot \phi$. However, saying that classical logic makes a substantive demand is not to say that the demand is not met. Say that a state *w* is a *world* if it is consistent and for every state *s* either *w* contains *s* or $s \sqcup w$ is inconsistent. Fine then showed that the theorems of classical logic are *world-valid*; that is, every theorem of classical logic is inexactly verified by each world. Say that a space of states is *thoroughly classical* if every state is either inconsistent or contained in a world. The demand that classical logic imposes on reality is, then, that the space of states is thoroughly classical.

The idea that the truths of intuitionistic logic has some special status not shared by the truths of classical logic, is hardly novel; but the idea is usually cashed out in epistemic or semantic terms—see, e.g., Dummett 1991 and Tennant 1996. What truthmaker semantics provides is a metaphysical interpretation of the difference between classical and intuitionistic logic. How a proposition is made true is a worldly matter, having nothing to do either with how we can know the proposition or with the meaning of the sentences expressing the proposition.^6^$$^{,}$$^7^

As we develop the truthmaker semantics for modal logic, we will see that some standard modal principles are null-valid whereas others are merely world-valid.

### Digression: Classical Content Intuitionistically Construed?

One could object that the theorems of classical logic only look substantial because we have construed their content intuitionistically. In particular, a classical logician could object to the intuitionistic treatment of negation.^8^

The most common approach to negation in the literature on truthmaking is *bilateralist*: one identifies a proposition *P* with the sets of its truthmakers and falsemakers. One then takes the truthmakers of $\lnot P$ to be the falsemakers of *P* and one takes the falsemakers of $\lnot P$ to be the truthmakers of *P*. Unlike on the intutionist view, the proposition *P* is identical to the proposition $\lnot \lnot P$.

However, while the classical logician disagrees with the intuitionist about negation, there is nothing in the bilateral approach that stops the classical logician from defining the intuitionistic conditional. The clause for the truthmakers for conditionals is as above. Once one works in a bilateral setting one also has to decide on the falsemakers for conditionals. A quite natural approach would take the falsemakers for $P \rightarrow Q$ to be fusions of truthmakers for *P* with falsemakers for $\lnot Q$.^9^ If one adopts the bilateral approach, the conditional $\lnot \lnot P \rightarrow P$ will thus be null-verified and so insubstantial.

However, it does not follow that all the theorems of classical logic are insubstantial. In particular, there is still no reason to accept that all instances of excluded middle are verified by the null state. Indeed, given that the classical logician accepts the standard account of the truthmakers for disjunctions, holding that all instances of excluded middle are null-verified would commit one to the fatalistic view that every proposition is either necessarily true or necessarily false.^10^

This possibility raises an interesting technical question. Consider the propositional language with $\wedge , \vee , \lnot , \rightarrow$ as its sole connectives. Let us consider truthmaker interpretations of this language with the standard bilateral clauses for conjunction, disjunction, and negation, and in addition the bilateral clauses for the conditional sketched above. What sentences are then null-valid? We have to leave exploration of this question and the extension of this bilateral framework to the modal setting to another occasion.

## Neighborhood Models

Let us adopt a naïve approach and simply assign to a state the set of propositions that it makes necessary; we thus obtain a truthmaker analogue of neighborhood semantics.^11^ (In §4 we will consider whether we can obtain a more illuminating formulation using accessibility relations). More formally, we assume that we have a partial function *N* that assigns sets of propositions to states. We must impose some conditions on *N*.

It is important to allow *N* to be partial. Consider, e.g., the modality “it is known by Williamson that”. The state of a particular pebble’s being on the beach is not a state that, as a whole, makes Williamson know anything. However, if Williamson comes to know that the pebble is on the beach, the state of the pebble’s being on the beach is plausibly part of the state that constitutes his knowledge that it is on the beach.^12^

The states on which *N* is defined are the *modal* states. While not every state is modal we will assume that a fusion of modal states is itself modal^13^:

(Modal Closure): If for all $i \in I$ the state $s_i$ is modal, then $\bigsqcup _{i \in I} s_i$ is modal

The next principle concerns the relationship between the propositions made necessary by a state and the propositions made necessary by a state containing the first state. The thought behind this monotonicity principle is that while the larger state may say more about what is necessary, what it says is necessary had better contain what the smaller state says is necessary.

To state this precisely, we introduce some standard terminology (Fine 2017a, b).

If *P*, *Q* are propositions say that *P* is *a conjunctive part of* *Q* (*Q* *contains* *P*) iff

(i) for all $s \in P$ there is $t \in Q$ such that $s \sqsubseteq t$; and
(ii) for all $s \in Q$ there is $t \in P$ such that $t \sqsubseteq s$.

Abusing notation we write $P \sqsubseteq Q$ for this notion.

Say that *P* *entails* *Q* (or *P* is a disjunctive part of *Q*) if $P \subseteq Q$. *P* inexactly entails *Q* iff for all $s \in P$ there is $t \sqsubseteq s$ such that $t \in Q$. More generally, *Q* is inexactly entailed by $P_0, P_1, \dotsc$ if for all $s_0 \in P_0, s_1 \in P_1, \dotsc$ there are $t_0 \sqsubseteq s_0, t_1 \sqsubseteq s_1, \dotsc$ such that $\bigsqcup \left\{ t_0, t_1, \dotsc \right\} \in Q$. We write $P \Vdash Q$ if *Q* is entailed by *P* and $P \mathrel {\Vdash _{\text {i}}}Q$ if *Q* is inexactly entailed by *P*. Finally, say that $P_0, P_1, \dotsc$ *weakly ground* *Q* (writing this $P_0, P_1, \dotsc \le Q$) iff for all $s_{0} \in P_0, s_1 \in P_1, \dotsc$ we have $\bigsqcup \left\{ s_0, s_1, \dotsc \right\} \in Q$.^14^

(Monotonicity): If $s \sqsubseteq t$ and both are modal then for all $P \in N(s)$ there is $Q \in N(t)$ such that $P \sqsubseteq Q$ and for all $P \in N(t)$ there is $Q \in N(s)$ such that $Q \sqsubseteq P$

To motivate the next constraint consider the following principle.

($$\Box _{\text {Down}}$$)
$$\Box (P \wedge Q)$$ inexactly entails $$\Box P$$

This principle appears unimpeachable: how could one make it necessary that $P \wedge Q$ without, in part, making it necessary that *P*? In order to ensure that ($\Box _{\text {Down}})$ holds we impose the principle

($$\hbox {CN}^{+}$$)
If $P \in N(s)$ and $Q \sqsubseteq P$ then there is $t \sqsubseteq s$ such that $Q \in N(t)$

This principle simply says that if a state makes a proposition necessary, then every proposition contained in that proposition is made necessary by some part of the state. (Note that the part need not be proper.)

The next two principles concern the structure of *N*(*s*). The first principle ensures that a state that makes the conjuncts necessary also make the conjunction necessary.

(Ground Closure): If $P_i \in N(s)$ for all $i \in I$ and $\left\{ P_i\right\} _{i \in I}$ ground *Q* then $Q \in N(s)$

Together (CN$^{+}$) and (Ground Closure) ensure that $\Box (\phi \wedge \psi )$ inexactly entails each of $\Box \phi$ and $\Box \psi$ and that $\Box \phi , \Box \psi$ together ground $\Box (\phi \wedge \psi )$.

Since we are working with exact truthmaker semantics, we do not expect that a proposition contained in a proposition made necessary by a state is itself made necessary by that state; however, if the proposition itself contains a proposition that is made necessary by the state, then it is natural to assume this. This gives us:

(Convexity): If $P, Q \in N(s)$ and $P \sqsubseteq R \sqsubseteq Q$ then $R \in N(s)$

So far we have not said anything about which states are modal. The final two principles deal with this. These principles say that $0$—the null state—and all the contradictory states are modal.

(Nullity): $N(0)$ is defined.
(Contradictory): If $c \in C$ then *N*(*c*) is defined and there is $C_{0} \subseteq C$ such that $\emptyset \ne C_0$, and $C_0 \in N(c)$

By Fine’s results we know that all theorems of intuitionistic propositional logic are made true by the null state and are in that sense insubstantial. In a normal modal logic the necessitation of a theorem is itself a theorem. So if we want the theorems of intuitionistic modal logic to be insubstantial in the same way, the null state has to be modal.^15^

To capture the rule of *ex falso* we have required that if *P* is a proposition then if *c* is contradictory then *P* is made true by a part of *c*. (Contradictory) ensures that the necessity of a proposition has this feature if the proposition does.

Extend the language of intuitionistic propositional logic with a necessity operator $\Box$ in the obvious way. An intuitionistic modal logic is any subset of $\mathcal {L}_{\Box }$ that contains intuitionistic propositional logic, is closed under modus ponens, substitution and the regularity rule $\phi \rightarrow \psi / \Box \phi \rightarrow \Box \psi$. **IntK**$_{\Box }$ is the smallest intuitionistic modal logic that contains the axiom $\Box \top$ as well as the distribution axiom

(**K**$$_{\Box }$$)
$$\Box (\phi \wedge \psi ) \leftrightarrow \Box \phi \wedge \Box \psi $$

An *exact modal model* consists of a set *S*, a parthood relation $\sqsubseteq$ on *S*, a subset $C \subseteq S$ of contradictory states, a function *N* satisfying the conditions above, as well as an interpretation function ${\llbracket \phantom {x} \rrbracket }$ assigning a proposition to each sentence. In addition to the conditions on ${\llbracket \phantom {x} \rrbracket }$ mentioned in §2 we require that ${\llbracket \Box \phi \rrbracket } = \left\{ s :{\llbracket \phi \rrbracket } \in N(s)\right\}$.

One can show—see Theorem A.10 below—that all the theorems of $\textbf{IntK}_{\Box }$ are null-valid. The insubstantiality of intuitionistic propositional logic extends to $\textbf{IntK}_{\Box }$.^16^

## Against Relational Semantics

What we have given above is a truthmaker analogue of neighborhood semantics. For *normal* modal logics, one can also provide a relational possible worlds semantics: the propositions necessary in a world are the propositions true in every world accessible from that world. Since $\textbf{IntK}_{\Box }$ is normal, it is natural to wonder whether one could also develop a relational truthmaker semantics. If the only desideratum was having a semantics with respect to which $\textbf{IntK}_{\Box }$ was sound and complete, a relational semantics would be adequate. There are, however, philosophical reasons to be unhappy with a relational semantics. This section presents that case.^17^

Modeling the approach on possible worlds semantics one takes there to be a relation *R* between states such that when *sRt*, then *t* is an exact truthmaker for every proposition that is made exactly necessary at *s*. We thus obtain the following analogue of the clause for possible worlds semantics.

($$\Box _{\text {Relational}}$$)
$s \Vdash \Box P$ iff $t \Vdash P$ for all *t* such that *sRt*

To make a relational account work we have to impose some constraints on the interaction of $\sqsubseteq$ and *R*. Say that a state *s* is *modal* if *sRt*, for some *t*. Since we want the propositions that are made necessary by a state to be contained in a proposition made necessary by the extensions of that state we have to impose the following analogue of (Monotonicity).

(Monotonicity$$_R$$)
If $$s \sqsubseteq s_0$$ are both modal and $$s_0 R t_0 $$ then there is $$t \sqsubseteq t_0$$ such that *sRt*

Without further constraints ($\Box _{\text {Down}}$) will not hold. To see the problem suppose that every *t* such that *sRt* is such that $t \Vdash P \wedge Q$. Any such *t* *inexactly* verifies *P*, but there is no reason to think that each such *t* *exactly* verifies *P*. Thus, if *s* exactly verifies $\Box (P \wedge Q)$ there is no reason to think that *s* *exactly* verifies $\Box P$. What is worse, there is no guarantee that *s* even *inexactly* verifies $\Box P$, which means that ($\Box _{\text {Down}}$) would fail.

One cannot respond to this challenge by changing ($\Box _{\text {Relational}}$) to say that *s* exactly verifies $\Box P$ iff *t* inexactly verifies *P* for all *t* such that *sRt*. That would collapse the distinction between exact and inexact truthmaking for necessitated propositions: any modal state extending an exact truthmaker for $\Box P$ would then itself be an exact truthmaker for $\Box P$.

What is required to ensure that ($\Box _{\text {Down}}$) holds is the Principle of Contained Necessities: if a proposition is made necessary by a state then any proposition contained in the aforementioned proposition is entailed by a proposition made necessary by a state contained in the aforementioned state. Formally, the following is necessary and sufficient to ensure ($\Box _{\text {Down}}$).

(CN): If *Q*, *P* are propositions such that $Q \sqsubseteq P$ and $\left\{ t :s R t\right\} \subseteq P$, then there is $s_0 \sqsubseteq s$ such that $\left\{ t :s_0 R t\right\} \subseteq Q$

It is easy to see that (CN) is sufficient for ($\Box _{\text {Down}}$). To see that it is necessary, suppose that *s* is a state and *P*, *Q* are propositions such that $Q \sqsubseteq P$, $\left\{ t :s R t\right\} \subseteq P$ but there is no $s_0 \sqsubseteq s$ with $\left\{ t :s_0 R t\right\} \subseteq Q$. By ($\Box _{\text {Relational}}$) $s \Vdash \Box P$. Since $Q \sqsubseteq P$, it is also the case that $P \subseteq P \wedge Q$, and thus $s \Vdash \Box (P \wedge Q)$. But by assumption there is no $s_0 \sqsubseteq s$ such that $s_0 \Vdash \Box Q$. We thus have a failure of ($\Box _{\text {Down}}$).^18^

There are thus certain situations that cannot be modeled by the relational approach. Let *P*, *Q* be two propositions such that no truthmaker for $P \wedge Q$ is a truthmaker for either *P* or *Q*. If a state *s* is an exact truthmaker for $\Box (P\wedge Q)$ then that state is not an exact truthmaker for $\Box P$ or $\Box Q$. Rather, what (CN) ensures is that *s* has a *proper* part that is an exact truthmaker for $\Box P$ and a (possibly different) proper part that is an exact truthmaker for $\Box Q$. On the neighborhood semantics of §3, on the other hand, we can simply assign a set containing the propositions $P,Q, P\wedge Q$ to the state *s*. Thus *s* can itself be an exact truthmaker for each of $\Box P, \Box Q$ and $\Box (P \wedge Q)$. The neighborhood semantics is thus more flexible than the relational semantics.

If this was merely an abstract possibility this would perhaps not be a significant strike against a relational semantics; however, this possibility is arguably often realized. In the next three subsections we present three scenarios, involving different modalities, and argue that they realize this abstract possibility.

### Legal Entanglement

If $$\Box $$ is interpreted as “it is law that” then the states *t* such that *sRt* are the states that are in (exact) conformity with the law. Sometimes what makes it a law that $$A \wedge B$$ is that a *single* act of legislation was passed by Congress (both houses), not vetoed by the President, and then finally not overturned by the Supreme Court. As a concrete example we may look at the Inflation Reduction Act (ira). Simplifying, the ira sets the tax credit for buyers of (certain) electric cars at $7500; it also caps the price of a month’s worth of insulin at $35.^19^ On plausible assumptions this situation cannot be modeled relationally.

Let *A* be the proposition that the tax credit is $7500. We understand this as the proposition that one’s tax bill is $7500 less than it otherwise would have been. Let *B* be the proposition that the price for insulin is at most $ 35, understanding this as the proposition that what you pay for the insulin is at most $35.^20^ Let *s* be the complex state that constitutes the ira being law. The state *s* makes it a law that $$A \wedge B$$. By ($$\Box _{\text {Relational}}$$) this means that every state *t* such that *sRt* is a fusion of a state making *A* true with a state making *B* true.

Clearly, the proposition *B* is contained in the proposition $$A \wedge B$$. (CN) thus requires that there is a part $$s_0$$ of *s* such that for all *t* if $$s_0 R t$$ then *t* is a truthmaker for *B*. Since the ira is a *single* piece of legislation there is no *proper* part of *s* that makes anything a law; so *s* itself has to make it a law that *B*. But this means that the states that exactly make it the case that one pays at most $35 for insulin also are states that exactly make it the case that one’s tax bill is $7500 less than it otherwise would have been. But this is implausible. An exact truthmaker for a proposition is a way for that proposition to obtain. One thus has to hold that a way for one to pay at most $35 for insulin is a way for one’s tax bill to be $7500 less than it otherwise would have been.^21^

### Epistemic Entanglement

Suppose $\Box$ is interpreted as “the agent knows that $\dots$”; then each state *t* such that *sRt* would be a state that exactly verifies the propositions the agent knows by being in state *s*. Suppose one sees two twins Alice and Allison screaming in the distance. One takes the scene in as a whole: one simply sees that they are *both* screaming, the state of seeing *them* scream is not composed of a distinct state of seeing that Alice screams and a distinct state of seeing that Allison screams. Rather, there is a single state of knowing that Allison screams which is also a state of knowing that Alice screams which is also a state of knowing that they both scream. There is no problem accommodating this on the neigborhood semantics of §3: one simple assigns to this state the propositions that Alice Screams, that Allison screams, as well as their conjunction. However, this causes a problem for ($\Box _{\text {Relational}}$). Suppose *s* is the state of one’s knowing that Alice and Allison scream. Then every state *t* such that *sRt* would be a fusion of two states one of which exactly verifies that Allison screams and the other of which exactly verifies that Alice screams. (CN) thus requires one to hold that the state verifying that Allison screams is the same state as the state verifying that Alice screams, but this is very implausible: it does not seem plausible that a way for Alice to scream is, in part, a way for Allison to scream.

Indeed, this case is not an outlier. For most propositions *P* one cannot know *just* *P* (and what is entailed by *P*): given one’s epistemic situation coming to know *P* might involve coming to know lots of propositions properly containing *P*. (Suppose that what makes one know that a tree is in front of one is one’s being in a certain visual state. But being in that visual state also makes one know, say, that the sun is shining from one’s upper right, that the leaves are green, that the light falls on the tree just so, $\dotsc$). The relational approach is ill-suited to model epistemic phenomena.

### Metaphysical Entanglement

The above cases involve non-metaphysical modalities; moreover, they are cases where there *could* be truthmakers for the necessities in accordance with (CN). After all, one may move closer to the sorry scene and see Alice’s and Allison’s screaming separately; and—though this is far-fetched—Congress could stop bundling different issues into a single bill for late night passage. However, there are arguably cases involving metaphysical modality where there could not be truthmakers satisfying (CN)—though these cases are admittedly not as clear as the legal and epistemic cases.

Consider a non-eliminative structuralist view of the natural numbers where the natural numbers are the results of applying an abstraction operation *A* to entries in $\omega$-sequences.^22^ Let us write *A*(*S*, *a*, *n*) to mean that *n* is the number that is abstracted from entry *a* in $\omega$-sequence *S*. It is plausible that the truthmakers for *En*—the proposition that *n* exists—are the truthmakers for propositions of the form *A*(*S*, *a*, *n*), where *a* is the *n*’th entry in the $\omega$-sequence *S*. A reasonable view about the nature of the abstraction operation *A* is that for each $\omega$-sequence *S*, if *a* is the *n*’th entry in *S*, then there is a truthmaker for *A*(*S*, *a*, *n*).

It is, moreover, plausible that the truthmakers for *A*(*S*, *a*, *n*) are distinct from the truthmakers for $A(S,a',n')$ whenever *a* and $a'$ are distinct entries in *S*. However, given the above view about the nature of the abstraction operation the truthmakers for *A*(*S*, *a*, *n*) and *A*(*S*, *b*, *m*) are necessarily connected in the sense that one obtains iff the other does.^23^ If that is right, one cannot just necessitate the existence of *n*; by necessitating the existence of one number one necessitates the existence of them all. This metaphysical view cannot be modeled relationally.

## Beyond IntK$_{\Box }$

Some modalities satisfy stronger principles than those of ${\textbf {IntK}}_{\Box }$. The modality “it is required that” satisfies the **D**-principle $\lnot \Box \bot$, which expresses that one is never required to do incompatible acts. Both “it is metaphysically necessary that” and “it is known that” satisfy the **T**-principle: $\Box \phi \rightarrow \phi$, expressing that what is necessary (known) is true. More contentiously, both knowledge and metaphysical modality are sometimes taken to satisfy the **4**-principle $\Box \phi \rightarrow \Box \Box \phi$, expressing that what is necessary (known) is necessarily necessary (known to be known). And metaphysical necessity is sometimes taken to satisfy the **B**-principle $\phi \rightarrow \Box \lnot \Box \lnot \phi$ expressing that what is the case is necessarily not impossible; and metaphysical necessity is often taken to satisfy the **5** principle $\lnot \Box \phi \rightarrow \Box \lnot \Box \phi$ expressing that what is not necessary is necessarily not necessary.

We have seen that the theorems of ${\textbf {IntK}}_{\Box }$ are null-valid. Should the principles that go beyond ${\textbf {IntK}}_{\Box }$ be null-valid or merely world-valid? Arguably, this turns out to depend on what modality we are considering.

Let us first consider the case of $\textbf{B}$—the axiom $P \rightarrow \Box \lnot \Box \lnot P$. Here null-validity is not appropriate. If $\textbf{B}$ is to be null-valid, then *P* has to inexactly entail $\Box \lnot \Box \lnot P$, and so any truthmaker for *P* has to contain a truthmaker for $\Box \lnot \Box \lnot P$. But there is simply no reason to think that a state that verifies *P* itself is a modal state or even contains a modal state that bears on the modal status of *P*. This point holds irrespective of how $\Box$ is interpreted, but it is perhaps especially clear for epistemic modality: the state of the pebble’s being on the beach need not contain any epistemic state of Williamson’s.^24^

Let us next turn to the **T**-principle. This is the only uncontroversial principle governing both the knowledge operator and the metaphysical necessity operator. To ensure that the *T*-axiom is null-valid one requires that if $P \in N(s)$, then there is some $t \sqsubseteq s$ such that $t \in P$. That is, one requires that a truthmaker for $\Box P$ be an inexact truthmaker for *P*: to make $\Box P$ necessary, one first, so to speak, makes *P* true and then one adds its necessity.

In the case of knowledge this seems correct: if Williamson knows that the pebble is on the beach this is partly because the pebble is on the beach. But in the case of metaphysical necessity one may balk. Does the necessity of its either raining or not raining partly consist in its raining? One might think not: in making it the case that *P* is necessary, one has not *thereby* made *P* the case.^25^

To see the worry it might help to think of the necessitated propositions as putting down requirements on what God has to do when creating reality. A state that makes it necessary that *P* requires of God that he include a verifier for *P* when he constructs reality, but a state that imposes such a requirement on God need not itself meet this requirement. If this is right we should just require that $\Box P \rightarrow P$ be inexactly verified at each world, that is, we should just require *world*-validity.^26^

This is a case where truthmaker semantics gives us something genuinely new: even though the **T**-principle holds for both metaphysical necessity and knowledge the *way* the principle is true differs between the two cases.^27^

A different phenomenon arises with the **4**-principle. For metaphysical necessity it might be reasonable to take the **4** principle to be null-valid.^28^ A natural way of ensuring this is by requiring that each modal state make itself necessary.^29^

(Self Necessity): If *N*(*w*) is defined, then there is $P \in N(w)$ such that $\left\{ w\right\} \sqsubseteq N(w)$

The $\textbf{4}$-principle is of course very contentious for knowledge, but even if one accepts that what is known is known to be known one cannot validate $\textbf{4}$ via (Self Necessity). Suppose Williamson knows that the pebble is on the beach and knows that he knows that the pebble is on the beach. Part of what makes him know that the pebble is on the beach is that the pebble is on the beach. But there is a very specific way the pebble is on the beach—it is in a specific location, partially covered by sand $\dotsc$.) But there is no plausibility to the claim that in knowing that the pebble is on the beach Williamson knows the exact location, the way it is partially covered, $\dotsc$.

How can we ensure the world-validity of principles like $\textbf{T}$ and $\textbf{B}$?

We begin by refining our understanding of what it is for a state space to be classical. If *w* is a world, let $m_w$ be the maximal modal state contained in *w*. (The existence of such a state is ensured by (Nullity) and (Modal Closure).) Say that a state-space is *modally classical* if it is classical and for all worlds *w* there is exactly one proposition $W \in N(m_w)$ and every $s \in W$ is a world.

The propositions that are made necessary at $m_w$ are the propositions that are made exactly true by each world. We then say that $\phi$ is a *world*-consequence of $\Gamma$ iff for every modally classical interpretation and every world *w*: if *w* inexactly verifies each $\gamma \in \Gamma$ then *w* inexactly verifies $\phi$. And we say that $\phi$ is *world-valid* iff $\phi$ is inexactly verified by every world in every modally classical interpretation.

It turns out that there are “purely logical” conditions that ensure that ${\textbf {T}}$, and **B**, and ${\textbf {5}}$ are true at each world. The details for how to ensure this are given in the appendix in table 3 but let us consider the case of **T** as an illustration. To ensure that every world that makes $\Box P$ true, makes *P* true as well it suffices that every state that makes $\Box P$ exactly true is incompatible with any state that makes $\lnot P$ exactly true. If that is the case, any world that contains a verifier for $\Box P$ will have to contain a verifier for *P* given that it cannot contain a verifier for $\lnot P$. The upshot of this is that while $\textbf{T}$ is not null-valid, the principle $\textbf{T}_{\lnot \lnot }$—that is, $\Box P \rightarrow \lnot \lnot P$—*is*.^30^

Above we noted that one can adopt intuitionistic truthmaker semantics without opposing classical logic. The theorems of classical logic are all true, but they are true in a more substantive way than the truths of intuitionistic logic. We have now seen that certain truths of modal logic—like ${\textbf {T}}$ and $\textbf{B}$—also might have substantive truthmakers.

## Conclusions and Further Work

This is obviously but the beginning of work in exact modal truthmaker semantics. Let us end by indicating some questions for future research. (While it would obviously be interesting to develop a truthmaker semantics for modal logic based on a bilateral treatment of negation, we restrict our attention to issues that arise using an intuitionistic treatment of negation.)

(i) It would be interesting to develop a truthmaker semantics with a primitive $\Diamond$ as well as a truthmaker semantics that takes both $\Diamond$ and $\Box$ as primitive. (See footnotes 24 and 29) This raises interesting questions about the relationship between $\lnot \Box P$ and $\Diamond \lnot P$.
  - Given the intuitionistic understanding of negation one should not expect $\lnot \Box P$ to inexactly entail $\Diamond \lnot P$. A truthmaker for $\lnot \Box P$ is merely a state that when fused with a truthmaker for $\Box P$ yields a truthmaker for $\bot$. But there does not seem to be any reason why a state that rules out that there is a truthmaker for the necessity of *P* should contain a state that makes *P* possible; in fact, there is no reason to assume that a truthmaker for $\lnot \Box P$ need be a modal state.
  - However, one should expect $\lnot \Box P$ to inexactly entail $\lnot \lnot \Diamond \lnot P$. Thus $\Diamond \lnot P$ is a world-consequence of $\lnot \Box P$. For the classical logician $\Box$ and $\Diamond$ would be, as it were, “world duals”.
  - Related to this point, Servi (1980) argued that if we take both $\Box$ and $\Diamond$ as primitive, then the intuitionistic analogue of classical ${\textbf {K}}$ is is the logic **FS**. This logic contains the axiom $\Box (\phi \rightarrow \psi ) \rightarrow (\Diamond \phi \rightarrow \Box \psi )$. It is not clear whether this principle should be taken to be null-valid or merely world-valid.
(ii) It is obviously of considerable interest to develop a truthmaker semantics for quantified modal logic. And the same holds for modal logic with *propositional* quantification. It is to be hoped that the finer resolution offered by truthmaker semantics might throw new light on the contingentism/necessitism debate (Williamson 2013).
(iii) Throughout we have assumed that *N*(*s*) satisfies (Ground Closure) but there are many applications where it is natural to relax this assumption. We mention two:
  - Suppose one reads $\Box P$ as someting like “Williamson *knows* and is *aware* that *P*”. One should not know all the consequences of what one knows, only the consequences of which one is aware. A natural restriction on (Ground Closure) is this. If one knows that *P*, *Q* is a logical consequence of *P*, and the *subject matter* of *Q* is contained in the subject matter of *P*, then one knows that *Q*.^31^
  - One might want to develop a truthmaker semantics for *essence* where for a state *s* to make *P* essentially true is for the state *s* to make *P* necessary and in addition for *s* to “contain” what the proposition *P* depends on.^32^
(iv) We have already defined the notion of weak ground, but we have only used it in the meta-language. An important task is to introduce an operator for weak full ground so that one can reason—in the object language—about the grounds for the propositions expressed in the language of intuitionistic modal logic.

This is work for a future occasion; hopefully, the results of the present paper show that there is much to be gained by that further work.

## A. Appendix

**Table 1:** Modal Axioms

**D**	$\lnot \Box \bot$
**T**	$\Box \phi \rightarrow \phi$
**T** $_{\lnot \lnot }$	$\Box \phi \rightarrow \lnot \lnot \phi$
$\textbf{B}$	$\phi \rightarrow \Box \lnot \Box \lnot \phi$
**B** $_{\lnot \lnot }$	$\phi \rightarrow \lnot \lnot \Box \lnot \Box \lnot \phi$
$\textbf{4}$	$\Box \phi \rightarrow \Box \Box \phi$
**5**	$\lnot \Box \phi \rightarrow \Box \lnot \Box \lnot \phi$
**5** $_{\lnot \lnot }$	$\lnot \Box \phi \rightarrow \lnot \lnot \Box \lnot \Box \lnot \varphi$

This appendix rigorously develops the account given in the text. Appendices A.1 to A.4 mainly rehearses material from Fine (2014). From appendix A.5 onwards we turn to modal matters.

### A.1 The Language of Intuitionistic Modal Logic

The language $\mathcal {L}_{\Box }$ of intuitionistic modal propositional logic is generated as follows. We have a countable infinity of atomic sentences $p_0,p_1, \dotsc$ and a designated sentence $\bot$. If $\phi , \psi$ are sentences then $\phi \wedge \psi$, $\phi \vee \psi$, $\phi \rightarrow \psi$, and $\Box \phi$ are sentences. We use $\lnot \phi$ as an abbreviation for $\phi \rightarrow \bot$, and we use $\phi \leftrightarrow \psi$ as an abbreviation for $(\phi \rightarrow \psi ) \wedge (\psi \rightarrow \phi )$. We use $\top$ for as an abbreviation for $p_0 \rightarrow p_0$. We use $\Gamma , \Delta , \dotsc$ for sets of sentences of $\mathcal {L}_{\Box }$.

### A.2 Normal Intuitionistic Modal Logic

An intuitionistic modal logic is any subset of $\mathcal {L}_{\Box }$ that contains intuitionistic propositional logic, is closed under modus ponens, substitution and the regularity rule $\phi \rightarrow \psi / \Box \phi \rightarrow \Box \psi$. **IntK**$_{\Box }$ is the smallest intuitionistic modal logic that contains the axiom $\Box \top$ as well as

(**K**$$_{\Box }$$): $$\Box (\phi \wedge \psi ) \leftrightarrow \Box \phi \wedge \Box \psi $$

We use the following naming convention. If $S_0, \dotsc , S_{n-1}$, $n \ge 0$ are some of the axioms in table 1 then **IntK**$\textbf{S}_1$ $\dots$ **S**$_m$ is the least intuitionistic modal logic containing **IntK**$_{\Box }$ and all instances of the axioms $S_0, \dotsc , S_{n-1}$. (Note that if $n=0$, then **IntK**${\textbf {S}}_0, \dotsc , {\textbf {S}}_{n-1}$ is **IntK**$_{\Box }$.) We write $\vdash \phi$ to mean that $\phi$ is in **IntK**$_{\Box }$ and we write $\vdash ^{\text {S}_1 \dotsc \text {S}_{\text {n}}} \phi$ to mean that $\phi$ is in the logic **IntK**$\textbf{S}_1\dotsc {\textbf {S}}_n$.

### A.3 State Spaces

A *state space* is a pair $\mathcal {S}= \langle S_{\mathcal {S}}, \sqsubseteq _{\mathcal {S}} \rangle$ where $S_{\mathcal {S}}$ is a set (intuitively of states) and $\sqsubseteq _{\mathcal {S}}$ is a complete partial order on $S_{\mathcal {S}}$. We write $\bigsqcup _{\mathcal {S}} S_0$ for the least upper bound of $S_0$ and refer to it as the fusion of $S_0$. We use $\sqcap _{\mathcal {S}} S_0$ for the greatest lower bound of $S_0$. If *s*, *t* are two states we write $s \sqcup _{\mathcal {S}} t$ for the fusion $\bigsqcup _{\mathcal {S}} \left\{ s, t\right\}$; similarly, we write $s_0 \sqcup _{\mathcal {S}} s_{1} \cdots \sqcup _{\mathcal {S}} s_{n-1}$ for the fusion of $\left\{ s_0, \dotsc , s_{n-1}\right\}$. Recall that by completeness $0_{\mathcal {S}}=\bigsqcup \emptyset$ exists.

When there is no risk of confusion we drop the subscript $\mathcal {S}$; for instance, we write $s \sqcup t$ instead of $s \sqcup _{\mathcal {S}} t$ and $0$ instead of $0_{\mathcal {S}}$.

By completeness, for any states *s*, *t* there is a state $s \dashrightarrow t= \sqcap \left\{ u :s \sqcup u \sqsupseteq t\right\}$. A space $\langle S, \sqsubseteq \rangle$ is *residuated* if for all *s*, *t* we have $(s \dashrightarrow t) \sqcup s \sqsupseteq t$. An *intuitionistic state space* is a triple $\mathcal {S}= \langle S_{\mathcal {S}}, \sqsubseteq _{\mathcal {S}}, C_{\mathcal {S}} \rangle$ where $\langle S_{\mathcal {S}}, \sqsubseteq _{\mathcal {S}}\rangle$ is a residuated state space and $C_{\mathcal {S}} \subseteq S_{\mathcal {S}}$ is non-empty set of states. Any state in *C* is said to be *contradictory*. We assume that *C* is complete in that if $\emptyset \ne C_{0} \subseteq C$ then $\bigsqcup C_{0} \in C$. The collection of *inconsistent* states is $C^+ = \left\{ t :c \sqsubseteq t, \text { for some } c \in C\right\}$. We say that *s*, *t* are *incompatible* if $s \sqcup t \in C^+$; they are *compatible* otherwise.

A set $P \subseteq S$ is *closed* if for all non-empty $P_0 \subseteq P$, $\bigsqcup P_0 \in P$; it is *convex* if whenever $s, t \in P$ and $s \sqsubseteq u \sqsubseteq t$, then $u \in P$; it is *regular* if it is both closed and convex. A *proposition* is simply a non-empty subset of *S*. An *intuitionistic proposition* is a proposition *P* such that for all $c \in C$ there is $c_0 \sqsubseteq c$ with $c_0 \in P$. From now on we use $P, Q, \dotsc$ (possibly with subscripts) to stand for (intuitionistic) propositions.

If *P* is a proposition and $s \in P$ we say that *s* is a *truthmaker* or *verifier* for *p* and write this $s \Vdash P$. If $s \Vdash P$ and $s \sqsubseteq t$ we say that *t* *inexactly* verifies (is an inexact truthmaker for) *P* and write this $t \mathrel {\Vdash _{\text {i}}}P$

If *P*, *Q* are (intuitionistic) propositions then the propositions $P \wedge Q$, $P \vee Q$, and $P \rightarrow Q$ are defined by the following^33^:

- $$P \wedge Q= \left\{ s_0 \sqcup s_1 :s_{0} \Vdash P \text { and } s_1 \Vdash Q\right\} $$.
- $$P \vee Q= \left\{ s :s \Vdash P \text { or } s \Vdash Q \right\} $$
- $$P \rightarrow Q =\Big \{t :t= \bigsqcup _{s \Vdash P} s \dashrightarrow f(s), \text { for some function }$$$$ f :P \rightarrow Q \Big \}$$

A *distinguished* state space (or *D*-space) is a tuple $\langle \mathcal {S}, \mathcal {R}\rangle$. Here $\mathcal {S} = \langle S, \sqsubseteq C \rangle$ is an intuitionistic state space and $\mathcal {R} \subset S$ is a designated set of states. (The “real” or “obtaining” states.) *R* satisfies the following:

Non-Vacuity: $\mathcal {R}$ is not empty
Consistency: $$\mathcal {R} \cap C= \emptyset $$
Part: If $s \sqsubseteq t$ and $t \in \mathcal {R}$, then $s \in \mathcal {R}$
Finite Fusion: If $s, t \in \mathcal {R}$ then $s \sqcup t \in \mathcal {R}$

We say that the *D*-space $\mathcal {S}$ is *closed* if it satisfies:

Closure: $$\bigsqcup \mathcal {R} \in \mathcal {R}$$

We say that it is *complete* if it satisfies

Completeness: For all $s \in S$ either $s \in \mathcal {R}$ or else *s* is incompatible with an $r \in \mathcal {R}$

A *D*-space is *classical* if it is both closed and complete.

If $\mathcal {S}$ is a state space and *w* is a consistent state say that *w* is a *world* if every state of $\mathcal {S}$ is either part of *w* or incompatible with *w*. If $\langle \mathcal {S}, \mathcal {R}\rangle$ is classical then there is a world *w* such that $\mathcal {R}= \left\{ s :s \sqsubseteq w\right\}$. A state space is *thorougly classical* if every consistent state is part of a world.

### A.4 Truth-maker semantics for intuitionistic propositional logic

An *exact model* for $\mathcal {L}$ is a tuple $\mathcal {E}=\langle \mathcal {S}_{\mathcal {E}}, {\llbracket \phantom {x} \rrbracket }_{\mathcal {E}} \rangle$. Here $\mathcal {S}_{\mathcal {E}}= \langle S, \sqsubseteq , C\rangle$ is an intuitionistic state space and ${\llbracket \phantom {x} \rrbracket }_{\mathcal {E}}$ is a function mapping the propositional letters of $\mathcal {L}$ to intuitionistic propositions over $\mathcal {S}$. An *exact designated model* is a tuple $\mathcal {E}=\langle \mathcal {S}, \mathcal {R}, {\llbracket \phantom {x} \rrbracket } \rangle$ where $\langle \mathcal {S}, {\llbracket \phantom {x} \rrbracket } \rangle$ is an exact model and $\langle \mathcal {S}, \mathcal {R}\rangle$ is a designated state space.

We extend ${\llbracket \phantom {x} \rrbracket }_{\mathcal {E}}$ to a function ${\llbracket \phantom {x} \rrbracket }_{\mathcal {E}}^{+}$ defined on all the sentences of $\mathcal {L}$ by recursion.

(i) $${\llbracket \bot \rrbracket }_{\mathcal {E}}^{+}= C$$;
(ii) $${\llbracket \phi \wedge \psi \rrbracket }_{{\mathcal {E}}}^{+} = {\llbracket \phi \rrbracket }_{{\mathcal {E}}}^{+} \wedge {\llbracket \psi \rrbracket }_{\mathcal {E}}^{+}$$;
(iii) $${\llbracket \phi \vee \psi \rrbracket }_{\mathcal {E}}^{+}= {\llbracket \phi \rrbracket }_{\mathcal {E}}^{+} \vee {\llbracket \psi \rrbracket }_{\mathcal {E}}^{+}$$;
(iv) $${\llbracket \phi \rightarrow \psi \rrbracket }_{\mathcal {E}}^+= {\llbracket \phi \rrbracket }_{\mathcal {E}}^{+} \rightarrow {\llbracket \psi \rrbracket }_{\mathcal {E}}^{+}$$

Whenever possible we will drop sub- and superscripts and write ${\llbracket \phantom {x} \rrbracket }$ for ${\llbracket \phantom {x} \rrbracket }_{\mathcal {E}}^{+}$.

When $\phi$ is a sentence of $\mathcal {L}$ and $\mathcal {E}=\langle \mathcal {S}, {\llbracket \phantom {x} \rrbracket }_{\rangle }$ is an exact model say that $\mathcal {E}, s \Vdash \phi$ iff $s \in {\llbracket \phi \rrbracket }$. $\mathcal {E}, s \mathrel {\Vdash _{\text {i}}}\phi$ is defined analogously. When $\mathcal {E}$ is clear from context, we may write $s \Vdash \phi$ (and similarly for $s \mathrel {\Vdash _{\text {i}}}\phi$). If $\mathcal {E}$ is a designated model with reality $\mathcal {R}$ then we say that $\phi$ is true in $\mathcal {E}$ iff $r \mathrel {\Vdash _{\text {i}}}\phi$ for some $r \in \mathcal {R}$.

Following Fine 2014, p. 569 we define the following notions of consequence:

#### Definition A.1

(i) $\Gamma \models _{i1} \phi$ iff for all models $\mathcal {E}$ and all states *s*, if $\mathcal {E}, s \mathrel {\Vdash _{\text {i}}}\gamma$ for all $\gamma \in \Gamma$, then $\mathcal {E},s \mathrel {\Vdash _{\text {i}}}\phi$.
(ii) $\Gamma \models _{i2} \phi$ iff for all models $\mathcal {E}$ and all consistent states *s*, if $\mathcal {E}, s \mathrel {\Vdash _{\text {i}}}\gamma$ for all $\gamma \in \Gamma$, then $\mathcal {E},s \mathrel {\Vdash _{\text {i}}}\phi$.
(iii) $\Gamma \models _{i3} \phi$ iff for all models $\mathcal {E}$ and the null state $0_{\mathcal {E}}$ of $\mathcal {E}$ if $\mathcal {E}, 0_{\mathcal {E}} \mathrel {\Vdash _{\text {i}}}\gamma$ for all $\gamma \in \Gamma$, then $\mathcal {E}, 0_{\mathcal {E}} \mathrel {\Vdash _{\text {i}}}\phi$.
(iv) Let *X* be a class of designated models; then $\Gamma \models _X \phi$ iff for all models $\mathcal {E}$ in *X* if $\gamma$ is true in $\mathcal {E}$ for all $\gamma \in \Gamma$, then $\phi$ is true in $\mathcal {E}$.

Write $\Gamma \vdash _I \phi$ to mean that $\phi$ is derivable from $\Gamma$ in some standard proof system for intuitionistic propositional logic; and let $\Gamma \vdash _C \phi$ mean that $\phi$ is derivable from $\Gamma$ in some standard proof system for classical propositional logic. Fine established the following soundness and completeness results.

#### Theorem A.2

(i) $\Gamma \models _{i1} \phi$ iff $\Gamma \models _{i2} \phi$ iff $\Gamma \models _{i3} \phi$ iff $\Gamma \vdash _I \phi$.
(ii) If *X* is the collection of closed or complete models then $\Gamma \models _X \phi$ iff $\Gamma \vdash _I \phi$;
(iii) If *X* is the collection of classical models then $\Gamma \models _X \phi$ iff $\Gamma \vdash _C \phi$.

Fine proved these results by relating exact (designated) models to the more familiar Kripke-models for intuitionistic logic. In proving soundness and completeness results for **IntK**$_{\Box }$ and stronger systems we will adopt a similar strategy. The next section, following Wolter and Zakharyaschev (1999), will present an (inexact) Kripke semantics for intuitionistic modal logic.

### A.5 Kripke semantics for intuitionistic modal logic

An *inexact modal space* (or *frame*) is tuple $\mathcal {K}= \langle K_{\mathcal {K}}, \le _{\mathcal {K}}, R_{\mathcal {K}}\rangle$. Here $K_{\mathcal {K}}$ is a set, $\le _{\mathcal {K}}$ is a partial order on $K_{\mathcal {K}}$, and $R_{\mathcal {K}}$ is an accessibility relation such that:

Mono$$_{\Box }^i$$: For all $$s_0$$ and $$ t_0$$: if $$s \le s_0, s_{0} R t_0$$, and $$t_0 \le t$$ then *sRt*

As usual, we say that a set $P\subseteq K$ is *upwards closed* if for all $s \in P$ and all *t* with $s \le t$ we have $t \in P$. An inexact or Kripke *model* is a tuple $\mathcal {K}= \langle K, \le , R, {\llbracket \phantom {x} \rrbracket } \rangle$, where $\langle K, \le , R \rangle$ is a frame and ${\llbracket \phantom {x} \rrbracket }_{\mathcal {K}} :\mathbb {P} \rightarrow \mathcal {P}(K)$ is a function taking each propositional letter *p* to an upwards closed set ${\llbracket p \rrbracket }_{\mathcal {K}}$ such that the following condition is satisfied:

Consistency: $${\llbracket \bot \rrbracket }_{\mathcal {K}}=\emptyset $$

When no confusion arises we drop subscripts writing just ${\llbracket p \rrbracket }$ instead of ${\llbracket p \rrbracket }_{\mathcal {K}}$.

We define what it is for a state *s* to *force* the truth of a sentence $\phi$ as follows, writing $\mathcal {K}, s \models \phi$ for “*s* forces $\phi$”.

#### Definition A.3

Let $\mathcal {K}=\langle K, \le , R, {\llbracket \phantom {x} \rrbracket }_{\mathcal {K}}\rangle$ be an inexact model and $s \in K$ be a state.

(i) $$\mathcal {K}, s \models p$$ iff $$s \in {\llbracket p \rrbracket } $$
(ii) $\mathcal {K}, s \models \phi \wedge \psi$ iff $\mathcal {K}, s \models \phi$ and $\mathcal {K}, s \models \psi$
(iii) $\mathcal {K}, s \models \phi \vee \psi$ iff $\mathcal {K}, s \models \phi$ or $\mathcal {K}, s \models \psi$
(iv) $\mathcal {K}, s \models \phi \rightarrow \psi$ iff for all $t \ge s$, if $\mathcal {K},t \models \phi$ then $\mathcal {K}, t \models \psi$
(v) $\mathcal {K}, s \models \Box \phi$ iff for all *t* such that *sRt* we have $\mathcal {K}, t \models \phi$

Note that it follows from (Consistency) that $\mathcal {K}, s \not \models \bot$ for all *s*. If $\mathcal {K}$ is clear from context we usually write $s \models \phi$ instead of $\mathcal {K}, s \models \phi$.

A straightforward induction establishes:

Heredity: If $s \models \phi$ and $s \le t$ then $t \models \phi$

As usual, we say that $\phi$ is a *Kripke-consequence* of $\Gamma$ ($\Gamma \models _{\mathcal {K}} \phi$) iff for all models $\mathcal {K}=\langle K, \le , R, {\llbracket \phantom {x} \rrbracket } \rangle$ and all $s \in K$, if $s \models \gamma$ for each $\gamma \in \Gamma$ then $s\models \phi$. We say that $\phi$ is *Kripke-valid* ($\models \phi$) if $\phi$ is a consequence of any set of sentences.

By imposing conditions on the accessibility relation *R* we can ensure the truth of various modal axioms. Some standard and not so standard axioms and the corresponding conditions are depicted in table 2.

**Table 2:** Inexact frame conditions

	Axioms	Conditions	*R* is
**D**	$\lnot \Box \bot$	$\exists t s R t$	Serial
**T**	$\Box \phi \rightarrow \phi$	*sRs*	Reflexive
**T**$_{\lnot \lnot }$	$\Box \phi \rightarrow \lnot \lnot \phi$	For all $s_{0} \ge s$ there is $s_1 \ge s_{0}$ and *t* such that	
		*sRt*, $s_{1} \ge t$	Weakly Reflexive
$\textbf{B}$	$\phi \rightarrow \Box \lnot \Box \lnot \phi$	$s R t \rightarrow \forall t_0 \ge t \exists s_0 (t_0 R s_0 \wedge s \le s_0)$	Symmetric$^{*}$
**B**$_{\lnot \lnot }$	$\phi \rightarrow \lnot \lnot \Box \lnot \Box \lnot \phi$	for all $s_0 \ge s$ there is $s_1 \ge s_{0}$ such that	
		for all $t, t_{0}$ such that $s_{1} R t$ and $t \le t_0$	
		there is $s_2 \ge s$ such that $t_0 R s_2$	Weakly Symmetric$^{*}$
$\textbf{4}$	$\Box \phi \rightarrow \Box \Box \phi$	$s R t \wedge t R u \rightarrow s R_{\Box } u$	Transitive
**5**	$\lnot \Box \phi \rightarrow \Box \lnot \Box \phi$	$s R t \rightarrow \exists s_0 (s \le s_0 \wedge \forall t_0 (s_0 R t_0 \rightarrow t R t_0)$	Euclidean$^{*}$
**5**$_{\lnot \lnot }$	$\lnot \Box \phi \rightarrow \lnot \lnot \Box \lnot \Box \phi$	for all $s_0 \ge s$ there is $s_1 \ge s_0$ and $s' \ge s$ such that	
		$\forall t \forall t_0 (s_1 R t \wedge s' R t_0 \rightarrow t R t_0)$	Weakly Euclidean$^{*}$

#### Theorem A.4

(Soundness)

(i) Every theorem of ${\textbf {IntK}}_{\Box }$ is true at every state in every model.
(ii) If a model satisfies a frame-condition from table 2 then the corresponding modal principle is true at every state in that model.

#### Proof

The proof is routine. But since they are unfamiliar will do the cases of ${\textbf {T}}_{\lnot \lnot }$, **B**, ${\textbf {B}}_{\lnot \lnot }$, and $\textbf{5}_{\lnot \lnot }$.

Let $$\mathcal {K}= \langle K, \le , R, {\llbracket \phantom {x} \rrbracket } \rangle $$ be a model where *R* is weakly reflexive and let *s* be a state. Suppose $$s \models \Box \phi $$ and let $$s \le s_{0}$$. Since *R* is weakly reflexive find $$s_1,t $$ such that $$s_{0} \le s_{1}$$, *sRt*, and $$t \le s_1$$. Since $$s \models \Box \phi $$, $$t \models \phi $$ and so $$s_1 \models \phi $$ by (Heredity). Since $$s_0$$ was arbitrary this shows that $$s \models \lnot \lnot \phi $$.

Suppose next that *R* is symmetric$^{*}$ and that $\mathcal {K}, s \models \phi$. Suppose *sRt* and let $t_0 \ge t$ be given. Since *R* is symmetric$^{*}$ find $s_0 \ge s$ such that $t_0 R s_0$. Since $s \models \phi$, $s_0 \models \phi$ and thus $t_0 \not \models \Box \lnot \phi$. Thus $t \models \lnot \Box \lnot \phi$ and so $s \models \Box \lnot \Box \lnot \phi$.

Suppose next that *R* is weakly symmetric$^{*}$ and suppose $\mathcal {K}, s \models \phi$. Let $s \le s_0$. Since *R* is weakly symmetric$^{*}$ find $s_1 \ge s_0$ witnessing weak symmetry$^{*}$. Let $t, t_0$ be such that $s_1 Rt$, and $t \le t_0$. Then there is $s_2 \ge s$ such that $t_0 R s_2$. Since $s \models \phi$ also $s_2 \models \phi$. But then $t_0 \not \models \Box \lnot \phi$ and so $t \models \lnot \Box \lnot \phi$. But then $s_1 \models \Box \lnot \Box \lnot \phi$ and so $s \models \lnot \lnot \Box \lnot \Box \lnot \phi$.

Suppose *R* is Euclidean$^{*}$. Suppose $s \models \lnot \Box \phi$ and let *sRt*. To show that $t \models \lnot \Box \phi$, let $t \le t_0$. By (Mono$_{\Box }^i$) $s R t_0$. Since *R* is Euclidean$^{*}$ there is $s_0 \ge s$ such that if $s_0 R u$ then $t_{0 } R u$. But since $s \models \lnot \Box \phi$, $s_0 \models \lnot \Box \phi$ which means that there is *u* such that $s_0 R u$ and $u \not \models \phi$. But then $t_0 \not \models \Box \phi$. And since $t_0$ was arbitrary $t \models \lnot \Box \phi$, and thus $s \models \Box \lnot \Box \phi$.

Finally, suppose that *R* is weakly Euclidean$^{*}$ and that $s \models \lnot \Box \phi$. To show that $s \models \lnot \lnot \Box \lnot \Box \phi$ let $s_0 \ge s$. Since *R* is weakly Euclidean$^{*}$ find $s_1 \ge s_0$ and $s' \ge s$ satisfying the conditions for being weakly Euclidean$^{*}$ and let $s_1 R t$. For each $t_0$ such $s' R t_0$ we have $t R t_0$. Since $s \models \lnot \Box \phi$, there is $t_0$ such that $s' R t_0$ and $t_0 \not \models \phi$. But then $t \not \models \Box \phi$. But then $s_1 \models \Box \lnot \Box \phi$. And thus $s \models \lnot \lnot \Box \lnot \Box \phi$, as desired. $\square$

#### Theorem A.5

(Completeness)

(i) If $\phi$ is true in every model of **IntK**$_{\Box }$ then $\vdash _{ \text {IntK}_{\Box }}\phi$.
(ii) If $\phi$ is true in every model satisfying the frame-conditions for axioms $S_{0} \dotsc S_n$ in table 2 then, $\vdash _{\text {IntKS}_{0} \cdots \text {S}_{\text {n}}} \phi$

The result is proved by a standard canonical model construction. Recall that a set of sentences $\Gamma$ is *prime* iff $\Gamma$ is closed under $\vdash$ and whenever $\phi \vee \psi \in \Gamma$, then $\phi \in \Gamma$ or $\psi \in \Gamma$. We say that $\Gamma \vdash \Delta$ iff $\vdash \bigwedge \Gamma _0 \rightarrow \bigvee \Delta _{0}$ for some finite subsets $\Gamma _0, \Delta _0$ of $\Gamma , \Delta$ respectively. (Similarly, for $\vdash _{S_1, \dotsc , S_n}$.)

The following lemma is proved in the standard way:

#### Lemma A.6

If $\Gamma \nVdash \Delta$, then there is a prime $\Gamma ' \supseteq \Gamma$ such that $\Gamma \nvdash \Delta$.

#### Proof of Theorem A.5

The canonical frame $\mathcal {C}=\langle K, \le , R \rangle$ is constructed as follows. The elements of *K* are the prime sets of sentences. If $\Gamma , \Delta \in K$ then $\Gamma \le \Delta$ iff $\Gamma \subseteq \Delta$. We set $\Gamma R \Delta$ iff $\phi \in \Delta$ for each $\phi$ such that $\Box \phi \in \Gamma$

Since some of the frame-conditions are unfamiliar we deal with the cases of $\textbf{T}_{\lnot \lnot }, {\textbf {B}}, {\textbf {B}}_{\lnot \lnot }$ and $\textbf{5}$.

We begin by showing that the canonical model for $\textbf{T}_{\lnot \lnot }$ is weakly reflexive. Let $\Gamma _0 \subseteq \Gamma _1$. We claim that there is prime $\Delta _0$ such that $\Gamma _0 R \Delta _0$ and $\Gamma _1 \cup \Delta _0$ is consistent. To begin let $\Delta _0'= \left\{ \phi :\Box \phi \in \Gamma _0\right\}$. Then $\Gamma _1 \cup \Delta '_0$ is consistent. For suppose otherwise, then there are $\phi _0, \dotsc , \phi _n \in \Delta _0'$ such that $\Gamma _1 \vdash \phi _0 \wedge \cdots \phi _n \rightarrow \bot$. But then also $\Gamma _1 \vdash \lnot \lnot \phi _0 \wedge \cdots \wedge \lnot \lnot \phi _n \rightarrow \bot$. But by ${\textbf {T}}_{\lnot \lnot }$ we have $\Box \phi _i \rightarrow \lnot \lnot \phi _{i} \in \Gamma _0 \subseteq \Gamma _1$ for each $i \le n$. And since $\Box \phi _i \in \Gamma _1$ for each $i \le n$ it follows that $\lnot \lnot \phi _i \in \Gamma _1$ for all *n*, and thus $\Gamma _1$ is inconsistent.

Since $\Gamma , \Delta _{0}'$ is consistent, $\Delta _0' \nvdash \Gamma ^{\lnot }$ where $\Gamma ^{\lnot }= \left\{ \lnot (\phi _0 \wedge \dotsc , \phi _m) :\phi _0, \dotsc , \phi _{m} \in \Gamma \right\}$. By Lemma A.6 we can extend $\Delta _{0}'$ to a prime $\Delta _{0}$ such that $\Delta _{0} \nvdash \Gamma ^{\lnot }$. It follows that $\Delta _{0}, \Gamma$ is consistent. This shows that *R* is weakly reflexive.

We next consider ${\textbf {B}}$. Suppose that $\Gamma R \Delta$ and let $\Delta \subseteq \Delta '$. We claim that $\Gamma '=\Gamma \cup \left\{ \delta :\Box \delta \in \Delta '\right\}$ is consistent. Suppose otherwise, then there are $\Box \delta _0, \dotsc , \Box \delta _n \in \Delta '$ and $\gamma _0, \dotsc , \gamma _n \in \Gamma$ such that $\vdash _{ {\textbf {B}}} \delta _{0} \wedge \cdots \wedge \delta _m \rightarrow \lnot (\gamma _0 \wedge \cdots \wedge \gamma _n )$. But then $\vdash _{ {\textbf {B}}} \Box (\delta _0 \wedge \cdots \delta _n) \rightarrow \Box \lnot (\gamma _{0} \wedge \cdots \wedge \gamma _{n})$. However since $\gamma _0 \wedge \cdots \wedge \gamma _n \in \Gamma$ we have $\Box \lnot \Box \lnot (\gamma _0 \wedge \cdots \wedge \gamma _n) \in \Gamma$ and thus $\lnot \Box \lnot (\gamma _0 \wedge \cdots \wedge \gamma _n ) \in \Delta$ and so $\lnot \Box (\delta _0 \wedge \cdots \wedge \delta _m ) \in \Delta \subseteq \Delta '$. But then $\Delta '$ is inconsistent. Contradiction. By Lemma A.6 we can extend $\Gamma '$ to a prime $\Gamma ''$. This shows that *R* is symmetric$^{*}$.

We next deal with $\textbf{B}_{\lnot \lnot }$. Let $\Gamma \subseteq \Gamma _0$. Let $\Gamma _1= \Gamma _0 \cup \left\{ \Box \lnot \Box \lnot \phi :\phi \in \Gamma \right\}$. Clearly, $\Gamma _1$ is consistent. Without loss of generality we may assume that $\Gamma _1$ is prime. Let $\Gamma _{1} R \Delta$ and let $\Delta \subseteq \Delta '$. We claim that $\Gamma '=\Gamma \cup \left\{ \delta :\Box \delta \in \Delta '\right\}$ is consistent. Suppose otherwise, then there are $\Box \delta _0, \dotsc , \Box \delta _n \in \Delta '$ and $\gamma _0, \dotsc , \gamma _n \in \Gamma$ such that $\vdash _{ {\textbf {B}}_{\lnot \lnot }} \delta _{0} \wedge \cdots \wedge \delta _m \rightarrow \lnot (\gamma _0 \wedge \cdots \wedge \gamma _n )$. But then $\vdash _{ {\textbf {B}}_{\lnot \lnot }} \Box (\delta _0 \wedge \cdots \delta _n) \rightarrow \Box \lnot (\gamma _{0} \wedge \cdots \wedge \gamma _{n})$. However, since $\gamma _0 \wedge \cdots \wedge \gamma _n \in \Gamma$ we have $\lnot \lnot \Box \lnot \Box \lnot (\gamma _0 \wedge \cdots \wedge \gamma _n) \in \Gamma _1$ and thus $\lnot \Box \lnot (\gamma _0 \wedge \cdots \wedge \gamma _n ) \in \Delta$ and so $\lnot \Box (\delta _0 \wedge \cdots \wedge \delta _m ) \in \Delta \subseteq \Delta '$. But then $\Delta '$ is inconsistent. Contradiction. By Lemma A.6 we can extend $\Gamma '$ to a prime $\Gamma ''$. This shows that *R* is weakly symmetric$^{*}$.

Finally, we deal with $\textbf{5}$. Suppose that $\Gamma R \Delta$. Let $\Gamma '= \Gamma \cup \left\{ \Box \delta :\Box \delta \in \Delta \right\}$. We claim that $\Gamma '$ is consistent. For suppose otherwise, then there are $\gamma _0, \dotsc , \gamma _n \in \Gamma$ and $\Box \delta _0, \dotsc , \Box \delta _m \in \Delta$ such that $\vdash _{ {\textbf {5}}} \gamma _0 \wedge \cdots \wedge \cdots \gamma _n \rightarrow \lnot \Box (\delta _0 \wedge \cdots \delta _m)$. But then $\lnot \Box (\delta _0 \wedge \cdots \wedge \delta _m) \in \Gamma$ and so by ${\textbf {5}}$ also $\Box \lnot \Box (\delta _0 \wedge \cdots \wedge \delta _m) \in \Gamma$. But this contradicts that $\Gamma R \Delta$. We conclude that $\Gamma '$ is consistent. By Lemma A.6 we may extend $\Gamma '$ to a prime $\Gamma _1$. Suppose that $\Sigma$ is such that $\Gamma _1 R \Sigma$. Then $\gamma \in \Sigma$ for all $\Box \gamma \in \Gamma _1$. But then also $\delta \in \Sigma$ for all $\Box \delta \in \Delta$. This shows that $\Delta R \Sigma$ and thus that *R* is euclidean$^{*}$.

We let the canonical model be $\langle K, \le , R, {\llbracket \phantom {x} \rrbracket } \rangle$ where ${\llbracket \phantom {x} \rrbracket }$ is defined by setting $\Gamma \in {\llbracket p \rrbracket }$ iff $p \in \Gamma$ for atomic *p*. We prove in the usual way by induction that $\Gamma \models \phi$ iff $\phi \in \Gamma$. As an illustration we give the case of $\phi = \Box \psi$.

Suppose $\Box \psi \in \Gamma$ and suppose that $\Gamma R \Delta$. By definition of *R*, $\psi \in \Delta$. But then by the induction hypothesis $\Delta \models \psi$, and so $\Gamma \models \Box \psi$.

For the other direction, suppose $\Box \psi \notin \Gamma$. Let $\Delta _0=\left\{ \gamma :\Box \gamma \in \Gamma \right\}$. We claim that $\Delta _0 \nvdash \psi$. For suppose otherwise. Then there are $\gamma _0, \dotsc , \gamma _n \in \Delta _0$ such that $\vdash \gamma _0 \wedge \cdots \wedge \gamma _n \rightarrow \psi$. But $\vdash \Box \gamma _0 \wedge \cdots \wedge \Box \gamma _n \rightarrow \Box \psi$ by Regularity and (**K**$_{\Box }$). But since $\Box \gamma _0, \dotsc , \Box \gamma _{n} \in \Gamma$ this means that $\Box \psi \in \Gamma$; contradiction. By Lemma A.6 we can extend $\Delta _{0}$ to a prime $\Delta$ such that $\Delta \nvdash \psi$. Since $\Gamma R \Delta$, this shows that $\Gamma \not \models \Box \psi$. $\square$

### A.6 Exact Modal Models

An *exact modal space* is a tuple $\mathcal {M}= \langle \mathcal {S}, N \rangle$ where $\mathcal {S}= \langle S, \sqsubseteq , C\rangle$ is an intuitionistic state space and *N* is a partial function from *S* to propositions over *S* that satisfies (Modal Closure), (Nullity), (Contradictory), (Ground Closure), (Convexity), (Monotonicity), and (CN$^{+}$).

We note the following consequence of (Ground Closure).

#### Observation A.7

Suppose *N*(*s*) is defined, Then we have:

(i) There is a unique regular proposition $M_{s} \in N(s)$ such that if $P \in N(s)$ then $P \sqsubseteq M_{s}$.
(ii) If $u \in M_s$ and $u \sqsubseteq u_0$ then $u_0 \in M_s$
(iii) If $s \sqsubseteq t$ and *N*(*s*), *N*(*t*) are both defined then $M_{s} \sqsubseteq M_t$.

#### Proof

We define

$$\begin{aligned} M_s= \left\{ t :t = \bigsqcup _{i \in I} \left\{ t_i :t_{i} \in Q_i\right\} , \text { for some { I} such that } N(s)= \left\{ Q_i :i \in I\right\} \right\} \end{aligned}$$

By (Ground Closure) $M_{s} \in N(s)$. By definition $Q \sqsubseteq M_{s}$ for each $Q \in N(s)$. To show that $M_{s}$ is convex suppose that $t,v \in M_{s}$ and $t \sqsubseteq u \sqsubseteq v$. Let $M' = (M_s {\setminus } \left\{ t\right\} ) \cup \left\{ u\right\}$. Clearly, $M_s \sqsubseteq M'$, and $M' \sqsubseteq M_s$, but then $M' \in N(s)$ by (Convexity). Let *I* be such that $t = \bigsqcup _{i \in I} t_i$. For all $i \in$ such that $Q_i \ne M'$ let $t_i'=t_i$; when $Q_i = M'$ let $t_i' = u$. Clearly, $u= \bigsqcup _{i \in I} t_i'$. This shows that $u \in M_s$.

To show that $M_{s}$ is closed it suffices to show that $\bigsqcup M_s \in M_s$. By (Ground Closure) every proposition weakly grounded by $M_s, M_s, \dotsc$ is in *N*(*s*), but any such proposition is contained in $M_s$.

For the second part suppose that $u \in M_s$ and $u \sqsubseteq u_0$. By (Ground Closure) $M_s \cup \left\{ u_{0}\right\} \in N(s)$. By part one $M_s \cup \left\{ u_0\right\} \sqsubseteq M_s$, but since $M_s$ is convex $u_0 \in M_s$.

For the third part, by (Monotonicity) there is $Q \in N(t)$ such that $M_{s} \sqsubseteq Q$. But $Q \sqsubseteq M_{t}$ by the first part. $\square$

An exact modal *model* is a tuple $\mathcal {E}= \langle \mathcal {S}_{\mathcal {E}}, {\llbracket \phantom {x} \rrbracket }_{\mathcal {E}}\rangle$ where $\mathcal {S}_{\mathcal {E}}$ is an exact modal state space and ${\llbracket \phantom {x} \rrbracket }_{\mathcal {E}}$ is an assignment of intuitionistic propositions to the atomic sentences.

We extend ${\llbracket \phantom {x} \rrbracket }$ to an assignment of propositions to all the sentences of $\mathcal {L}_{\Box }$ by recursion. The clauses are as in the propositional case, and in addition we have:

- $${\llbracket \Box \phi \rrbracket }_{\mathcal {E}}= \left\{ s :{\llbracket \phi \rrbracket }_{\mathcal {E}} \in N(s)\right\} $$

We need to check that ${\llbracket \phantom {x} \rrbracket }_{\mathcal {E}}$ always assigns intuitionistic propositions. This is established by the obvious induction, the only new case being $\Box \phi$. Suppose $c \in C$. By (Contradictory) let $C_{0} \subseteq C$ be non-empty such that $C_0 \in N(c)$. By the induction hypothesis, there is a function $f:C_0 \rightarrow S$ such that for all $c_{0} \in C_{0}$ we have $f(c_{0}) \sqsubseteq c_{0}$ and $f(c_0) \Vdash {\llbracket \phi \rrbracket }$. Thus $\left\{ f(c_0) :c_0 \in C_0\right\} \sqsubseteq C_0$. Thus by (CN$^{+}$) there is $c_1 \sqsubseteq c$ such that $\left\{ f(c_0) :c_0 \in C_0\right\} \in N(c_1)$; and so $c_1 \Vdash \Box \phi$. This shows that ${\llbracket \phi \rrbracket }_{\mathcal {E}}$ is an intuitionistic proposition.

**Table 3:** Exact frame conditions

	Modal axiom	
**D**	$\Box \phi \rightarrow \lnot \Box \lnot \phi$	If $P \in N(s)$ and $\lnot P \in N(t)$
		then $s \sqcup t$ is inconsistent
**T**	$\Box \phi \rightarrow \phi$	$\exists t (t \in M_s \wedge t \sqsubseteq s )$
**T**$_{\lnot \lnot }$	$\Box \phi \rightarrow \lnot \lnot \phi$	$P \in N(s) \wedge t \in \lnot P \rightarrow s \sqcup t \text { is inconsistent}$
$\textbf{B}$	$\phi \rightarrow \Box \lnot \Box \lnot \phi$	for all *s* there is an *s*-proponent $m \sqsubseteq s$
$\textbf{B}_{\lnot \lnot }$	$\phi \rightarrow \lnot \lnot \Box \lnot \Box \lnot \phi$	For all *s*, if *m* is an *s*-opponent, then *s* and *m* are incompatible
$\textbf{4}$	$\Box \phi \rightarrow \Box \Box \phi$	If $v \in P$ and $P \in N(w)$ then
		$$\exists v_0 \sqsubseteq v (P \in N(v_{0}))$$
$\textbf{5}$	$\lnot \Box \phi \rightarrow \Box \lnot \Box \phi$	For all *s* and all $t \in M_s$, there is $s_0 \sqsupseteq s$ such that $M_t \sqsubseteq M_{s_0}$
		if *t* is consistent there is consistent such $s_0$
$\textbf{5}_{\lnot \lnot }$	$\lnot \Box \phi \rightarrow \lnot \lnot \Box \lnot \Box \phi$	For all $s\sqsubseteq s_0$ there is consistent $s_{1} \sqsupseteq s_0$ such that for all $t \in M_{s_1}$ there is $s' \sqsupseteq s$
		such that $M_t \sqsubseteq M_{s'}$; if *t* is consistent, there is consistent such *s*

The conditions required on *N* for validating various modal principles are depicted in table 3. We require the following definitions. If *t* is a state, let $m_{t}$ be the largest modal state contained in *t* and put $M_t= M_{m_t}$. If *s* is a state we use $\lnot \left\{ s\right\}$ for the proposition $\left\{ s \dashrightarrow c :c \in C\right\}$. Say that *t* is an *s*-*guardian* iff whenever $\lnot \left\{ s\right\} \sqsubseteq M_{u}$ then *t* is incompatible with *u*. Say that *m* is an *s*-*proponent* iff all $t \in M_m$ are *s*-guardians. (We write $\text {Pro}(s)$ for the set of *s*-guardians.) What is required for ${\textbf {B}}$ is thus that every state *s* contains an *s*-proponent.

Say that *m* is an *s*-*opponent* iff

$$\begin{aligned} m=\bigsqcup _{u \in \text {Pro}(s), f :\text {Pro}(s) \rightarrow C} u \dashrightarrow f(u) \end{aligned}$$

An *s*-opponent is thus a state that “knocks out” each *s*-proponent. What is required for ${\textbf {B}}_{\lnot \lnot }$ is thus that every states is incompatible with each *s*-opponent.

### A.7 Transforming exact to inexact models

If $\mathcal {E}= \langle S, \sqsubseteq , C, N, {\llbracket \phantom {x} \rrbracket } \rangle$ is an exact modal model we associate it with an inexact modal model $\mathcal {K}/\mathcal {E}= \langle K_{\mathcal {K}/\mathcal {E}}, \le _{\mathcal {K}/\mathcal {E}}, R_{\mathcal {K}/\mathcal {E}}, {\llbracket \phantom {x} \rrbracket }_{\mathcal {K}/\mathcal {E}}\rangle$ as follows.

- *K* is the set of consistent states from *S*;
- $\le$ is the restriction of $\sqsubseteq$ to *K*;
- $R \subseteq K \times K$ is defined by *sRt* iff $t \in M_s$. (Recall that we use $M_s$ to mean $M_m$, where *m* is the largest modal state contained in *s*.)
- $${\llbracket p \rrbracket }_{\mathcal {K}/\mathcal {E}}=\left\{ s \in K :\exists t ( t \in {\llbracket p \rrbracket } \wedge t \sqsubseteq s)\right\} $$

#### Theorem A.8

(i) $\mathcal {K}/\mathcal {E}$ is a Kripke-model
(ii) If *N* satisfies a condition in table 3 then *R* satisfies the corresponding condition in table 2.

#### Proof

We check that $\mathcal {K}/{\mathcal {E}}$ is an inexact model. By definition ${\llbracket \phantom {x} \rrbracket }_{\mathcal {K}/\mathcal {E}}$ assigns an upwards closed set to each propositional letter. To see that (Mono$_{\Box }^i$) holds suppose first that $s \le s_0$ and $s_{0} R t$. By definition of *R* we have $t \in M_{s_0}$. By Observation A.7$M_{s} \sqsubseteq M_{s_0}$ thus there is $t_1 \in M_{s}$ such that $t_1 \sqsubseteq t$ and thus *sRt*, since $M_s$ is upwards closed. If *sRt* and $t \le t_0$ then $s R t_0$ follows because $M_s$ is upwards closed.

For the second part, we take the conditions in order.

$\textbf{D}$. Let *s* be arbitrary and consistent and let $s_{0} \sqsubseteq s$ be the maximal modal state contained in *s*. If $M_{s_0} \subseteq C^{+}$ then $P \wedge \lnot P \in N(s_{0})$, for each *P* by (Contradictory); thus $s_0$ is inconsistent since *N* satisfies the **D**-condition. We can conclude that there is $t \in M_{s_0}$ with $t \in K$.

**T**. Let *s* be arbitrary and $m \sqsubseteq s$ be maximal modal. Since *N* satisfies the ${\textbf {T}}$-condition, there is $t \in M_m$ with $t \sqsubseteq m$. Since $m \sqsubseteq s$ it follows that *sRs*.

$\textbf{T}_{\lnot \lnot }$. Let $s, s_0 \in K$ be arbitrary with $s \sqsubseteq s_0$ and let $m \sqsubseteq s$ be maximal modal. Observe first that $M_m \cap K \ne \emptyset$. For otherwise, $M_m \subseteq C^+$ in which case $0 \in \lnot M_m$ and so $m= m \sqcup 0$ would have to be inconsistent by the $\textbf{T}_{\lnot \lnot }$-condition. Thus there is *t* such that *sRt*. Suppose that $s_0 \sqcup t$ is inconsistent for each *t* such that *sRt*. Then in particular $s_{0 } \sqcup t$ is inconsistent for each $t \in M_m$. But then $s_0 \mathrel {\Vdash _{\text {i}}}\lnot M_m$, and so, by (CN$^{+}$), there is $u \sqsubseteq s_0$ such that $u \in \lnot M_{m}$. Since *N* satisfies the $\textbf{T}_{\lnot \lnot }$-condition $s \sqcup u$ is inconsistent, and so $s_0$ is inconsistent. Contradiction. We conclude that there is $s_1 \ge s_0$ with $s_{1} \ge t$ for some *t* such that *sRt*.

$\textbf{B}$. Suppose $s, t, t_0 \in K$ such that *sRt* and $t \le t_{0}$. Let $m \le s$ be maximal modal; by definition of *R* we have $t \in M_m$. Let $m_0 \le t_{0}$ be maximal modal. Suppose that there is no $s_0 \in K$ with $s_0\ge s$ and $s_{0} \ge u$ for some $u \in M_{m_0}$. Then for all $u \in M_{m_0}$ we have that $s \sqcup u$ is inconsistent. Thus $\lnot \left\{ s\right\} \sqsubseteq M_{m_0}$. Since $t_0$ is not inconsistent $m_0 \sqcup t$ is not inconsistent, but this contradicts that *N* satisfies the $\textbf{B}$-condition.

$\textbf{B}_{\lnot \lnot }$. Suppose $s, s_0$ are both in *K* and $s \le s_0$. We need to show that there is $s_{1} \ge s_0$ such that if $m \sqsubseteq s_1$ is maximal modal, then *m* is an *s*-proponent.

Consider the set of all *s*-proponents. If this is the empty set, then the null state is an *s*-opponent. But then $s \sqcup 0$ is inconsistent by the ${\textbf {B}}_{\lnot \lnot }$ condition; since $s \in K$ this is impossible. Suppose that for no $m \in \text {Pro}(s)$ is $s_0 \sqcup m$ consistent. Then there is $f :\text {Pro}(s) \rightarrow C$ such that $m \dashrightarrow f(s) \sqsubseteq s_0$ for each $m \in \text {Pro}(s)$, and so $s_0$ contains an *s*-opponent. But this is impossible since by the $\textbf{B}_{\lnot \lnot }$-condition *s* is incompatible with every *s*-opponent. We conclude that there is an *s*-proponent *m* such that $s_{1} \sqcup m$ is consistent. Let $m_1 \sqsubseteq (s_{1} \sqcup m)$ be maximal modal. Every $t \in M_{m_1}$ contains a $t' \in M_m$ and since every $t' \in M_m$ is an *s*-guardian, so is every $t \in M_{m_1}$.

$\textbf{5}$. Suppose *sRt*. Then *t* is consistent and $t \in M_s$. Since *N* satisfies the $\textbf{5}$-condition let $s_0 \ge s$ be consistent and such that $M_{t} \sqsubseteq M_{s_0}$. But $M_t$ is upwards closed so it follows that if $s_0 R u$ then *tRu* and so *R* is euclidean$^{*}$.

**5**$$_{\lnot \lnot }$$. Let $$s_0 \ge s$$; by the $${\textbf {5}}_{\lnot \lnot }$$-condition find $$s_1 \ge s_{0}$$ such that for all consistent $$t \in M_{s_1}$$, there is consistent $$s' \sqsupseteq s$$ with $$M_t \sqsubseteq M_{s'} $$. But then $$s_1 $$ is such that for all *t* with $$s_{1} R t$$ we have $$s' \ge s$$ such that for all *u* with $$s' R u$$ we have *tRu*. This show that *R* is weakly euclidean$$^{*}$$. $$\square $$

We next establish the following.

#### Theorem A.9

Let $\mathcal {E}=\langle E, \sqsubseteq , C, N, {\llbracket \phantom {x} \rrbracket } \rangle$ be an exact model and let $\mathcal {K}/\mathcal {E}=\langle K, \le , R, {\llbracket \phantom {x} \rrbracket }_{i}\rangle$ be the associated inexact model. Then for each $s \in K$ and each sentence $\phi$ we have:

$$\begin{aligned} \mathcal {E}, s \mathrel {\Vdash _{\text {i}}}\phi \Leftrightarrow \mathcal {K}/\mathcal {E}, s \models \phi \end{aligned}$$

#### Proof

By induction on the complexity of $\phi$. Except the case for $\Box$ the proof is as in Fine (2014), but we have included the proof for definiteness.

For atomic *p* the result is immediate by the definition of ${\llbracket \phantom {x} \rrbracket }_{\mathcal {K}/\mathcal {E}}$.

We have $\mathcal {E}, s \mathrel {\Vdash _{\text {i}}}\phi \wedge \psi$ iff there is $s_0 \sqsubseteq s$ such that $\mathcal {E}, s \Vdash \phi \wedge \psi$ iff there are $s_1, s_2 \sqsubseteq s$ such that $\mathcal {E}, s_1 \Vdash \phi$ and $\mathcal {E}, s_2 \Vdash \psi$ iff (by the induction hypothesis) there are $s_1, s_2 \sqsubseteq s$- such that $\mathcal {K}/\mathcal {E}, s_1 \models \phi$ and $\mathcal {K}/\mathcal {E}, s_2 \models \psi$ iff $\mathcal {K}/\mathcal {E}, s \models \phi \wedge \psi$.

We have $\mathcal {E}, s \mathrel {\Vdash _{\text {i}}}\phi \vee \psi$ iff there is $s_0 \sqsubseteq s$ such that $\mathcal {E}, s \Vdash \phi \vee \psi$ iff there is $s_0 \sqsubseteq s$ such that $\mathcal {E}, s_0 \Vdash \phi$ or $\mathcal {E}, s_0 \Vdash \psi$ iff (by the induction hypothesis) there is $s_0 \sqsubseteq s$ such that $\mathcal {K}/\mathcal {E}, s_0 \models \phi$ or $\mathcal {K}/\mathcal {E}, s_0 \models \psi$ iff $\mathcal {K}/\mathcal {E}, s \models \phi \wedge \psi$.

We have $\mathcal {E}, s \mathrel {\Vdash _{\text {i}}}\phi \rightarrow \psi$ iff there is $s_0 \sqsubseteq s$ such that $\mathcal {E}, s_0 \Vdash \phi \rightarrow \psi$. Find a function *f* such that for every verifier *t* of $\phi$, $t \dashrightarrow f(t) \sqsubseteq s_0$. Now let $s_1 \ge s$ be such that $\mathcal {K}/\mathcal {E}, s_1 \models \phi$. By the induction hypothesis $\mathcal {E}, s_1 \mathrel {\Vdash _{\text {i}}}\phi$ so let $s_2 \sqsubseteq s_1$ be such that $\mathcal {E}, s_2 \Vdash \phi$. We have that $\mathcal {E}, s_0 \sqcup s_2 \mathrel {\Vdash _{\text {i}}}\psi$ and so $\mathcal {E}, s_1 \mathrel {\Vdash _{\text {i}}}\psi$; by the induction hypothesis $\mathcal {K}/\mathcal {E}, s_1 \models \psi$. Thus $\mathcal {K}/\mathcal {E}, s \models \phi \rightarrow \psi$.

For the other direction suppose $\mathcal {K}/\mathcal {E}, s \models \phi \rightarrow \psi$. Let $s_0$ be arbitrary such that $\mathcal {E}, s_0\Vdash \phi$. If $s \sqcup s_0$ is inconsistent let $f(s_0)$ be some element of *C* contained in $s \sqcup s_0$. If $s \sqcup s_0$ is consistent, then $\mathcal {E}, s \sqcup s_0 \mathrel {\Vdash _{\text {i}}}\phi$ and so $\mathcal {K}/\mathcal {E}, s \sqcup s_0 \models \phi$ by the induction hypothesis. Thus $\mathcal {K}/\mathcal {E}, s \sqcup s_0 \models \psi$ and so $\mathcal {E}, s \sqcup s_0 \mathrel {\Vdash _{\text {i}}}\psi$. Let $f(s_0) \sqsubseteq s \sqcup s_0$ be such that $\mathcal {E}, f(s_0) \Vdash \psi$. We have $s_0 \dashrightarrow f(s_0) \sqsubseteq s$. Let $s_1 = \bigsqcup _{s_0 \in {\llbracket \phi \rrbracket } } s_{0} \dashrightarrow f(s_0)$. This shows that $\mathcal {E}, s \mathrel {\Vdash _{\text {i}}}\phi \rightarrow \psi$.

Suppose that $\mathcal {E}, s \mathrel {\Vdash _{\text {i}}}\Box \phi$. Then let $s_0 \sqsubseteq s$ be such that $\mathcal {E}, s_{0} \Vdash \Box \phi$. Let *t* be arbitrary such that *sRt*. By definition of *R* we have $t \in M_s$. Since $s_0 \Vdash \Box \phi$, ${\llbracket \phi \rrbracket } \in N(s_0)$. By (Monotonicity) ${\llbracket \phi \rrbracket } \sqsubseteq M_s$. But then $\mathcal {E}, t \mathrel {\Vdash _{\text {i}}}\phi$ and so, by the induction hypothesis, $\mathcal {K}/\mathcal {E}, t \models \phi$. Since *t* was arbitrary this shows that $\mathcal {K}/\mathcal {E}, s \models \Box \phi$.

For the other direction, suppose that $\mathcal {K}/\mathcal {E}, s \models \Box \phi$. For each *t* such that *sRt* we have $\mathcal {K}/\mathcal {E}, t \models \phi$. By the induction hypothesis, $\mathcal {E}, t \mathrel {\Vdash _{\text {i}}}\phi$, for each *t* such that *sRt*. By definition of *R* we have $\left\{ t :s Rt\right\} = M_{s}$. For each $t \in M_s$ let $f(t) \sqsubseteq t$ be such that $\mathcal {E}, f(t) \Vdash \phi$. Clearly $\left\{ f(t):t \in M_s\right\} \sqsubseteq M_{s}$. Thus by (CN$^{+}$) find $s_0 \sqsubseteq s$ such that $\left\{ f(t) :t \in M_{s}\right\} \in N(s_0)$. $\mathcal {E}, s_0 \Vdash \Box \phi$ and thus $\mathcal {E}, s \mathrel {\Vdash _{\text {i}}}\Box \phi$. $\square$

#### Theorem A.10

(Soundness) If $\Gamma \vdash ^{{ \text {S}_{1},\, \dotsc \, \text {S}_{{n}}}} \phi$ then $\Gamma \Vdash ^{{ \text {S}_1, \dotsc , \text {S}_n}}_{i123} \phi$.

#### Proof

Suppose that $\Gamma \nVdash ^{ {S}_1, \dotsc , {S}_n}_{i123} \phi$ and let $\mathcal {E}, s$ be such that $\mathcal {E},s \mathrel {\Vdash _{\text {i}}}\Gamma$ but . We may take *s* to be consistent. By Theorem A.9 we have $\mathcal {K}/\mathcal {E}, s \models \Gamma$ but $\mathcal {K}/\mathcal {E}, s \not \models \phi$. Moreover, $\mathcal {K}/\mathcal {E}$ satisfies the conditions $\text {S1}, \dotsc , \text {Sn}$ iff $\mathcal {E}$ satisfies the conditions $\text {S1}, \dotsc , \text {Sn}$. But we know that $\vdash$ is sound with respect to $\models ^{S_1, \dotsc , S_n}$; the result follows. $\square$

### A.8 Transforming inexact models to exact models

We now wish to prove a completeness theorem with respect to exact modal models. In the modal case we cannot show that if $\Gamma \Vdash _{i3} \phi$, then $\phi$ follows from $\Gamma$ in **IntK**$_{\Box }$. To see this, observe that $\phi \Vdash _{i3} \Box \phi$. For let $\mathcal {E}$ be any model and suppose $\mathcal {E}, 0\Vdash \phi$. Then by (Nullity), (CN$^{+}$), and (Ground Closure) ${\llbracket \phi \rrbracket }_{\mathcal {E}} \in N(0)$. And thus $\mathcal {E}, 0\mathrel {\Vdash _{\text {i}}}\Box \phi$. But clearly, we cannot derive $\Box \phi$ from $\phi$ in **IntK**$_{\Box }$.

However, we do have the following.

#### Theorem A.11

Completeness If $\Gamma \Vdash ^{S_1, \dotsc , S_n}_{i1,2} \phi$ then $\phi$ follows from $\Gamma$ in **IntK**$S_1 \dotsc S_n$.

In interesting exact models the interpretations of the propositional letters are not upwards closed; but there is nothing that precludes this. The completeness proof exploits this by transforming Kripke-models for intuitionistic modal logic to exact modal models. This strategy was used by Fine in his completeness proof for intuitionistic propositional logic; the modal case raises a number of complications.

#### Definition A.12

A Kripke-model $\mathcal {T}= \langle T, \le , R, {\llbracket \phantom {x} \rrbracket } \rangle$ is *tree-like* iff

(i) There is a unique $r \in T$ such that *T* is the set of nodes that can be reached from *r* by any combination of steps using $\le$ and *R*. Call this *r* the *root* of $\mathcal {T}$.
(ii) Whenever $s,t \in T$ are $\le$-incomparable then there is no *u* with $s, t \le u$;
(iii) Any strictly ascending infinite chain $s_0< s_1< s_2 < \dotsc$ in $\mathcal {T}$ is unbounded.

We will need the following results about the tree-like models.

#### Theorem A.13

Let $\mathcal {K}= \langle K, \le , R, {\llbracket \phantom {x} \rrbracket }_{\mathcal {K}}\rangle$ be a Kripke-model. Then there is a tree-like Kripke model $\mathcal {T}=\langle T, \le _{T}, R_{T}, {\llbracket \phantom {x} \rrbracket }_{\mathcal {T}}\rangle$ and an embedding $f :\mathcal {K} \rightarrow \mathcal {T}$ such that for all $s \in \mathcal {K}$

$$\begin{aligned} \mathcal {K}, s \models \phi \Leftrightarrow \mathcal {T}, f(s) \models \phi \end{aligned}$$

Moreover, if $\mathcal {K}$ satisfies any of the conditions in table 2 then so does $\mathcal {T}$.

#### Proof

We define the tree-like model $\mathcal {T}= \langle T, \le _{T}, R_{T}, {\llbracket \phantom {x} \rrbracket }_{\mathcal {T}}\rangle$ as follows.

We let $\mathcal {T}$ be the finite sequences $\bar{s}= (s_0, s_{1}, \dotsc , s_{n-1})$, $n \ge 0$ of nodes from *K* such that $s_0 \le s_1 \le \dotsc \le s_{n-1}$. Note that we allow the empty sequence (). We define the tree order $\le _T$ by saying that $\bar{s} \le _T \bar{t}$ iff $\bar{t}$ is an end-extension of $\bar{s}$.

The order $$R_T$$ is defined as follows. $$\bar{s} R_T (t_0, \dotsc , t_n)$$ iff there is $$(s_0, \dotsc , s_m)$$ such that $$\bar{s} \le _T (s_0, \dotsc , s_m) $$ and $$ s_m R t_n$$. If *R* is reflexive, we in addition require that $$() R_T ()$$.

We define ${\llbracket p \rrbracket }_{\mathcal {T}}$ by saying $(s_0, \dotsc , s_m) \in {\llbracket p \rrbracket }_{\mathcal {T}}$ iff $s_m \in {\llbracket p \rrbracket }_{\mathcal {K}}$. For the empty sequence we require $() \notin {\llbracket p \rrbracket }_{\mathcal {T}}$.

We have to check that (Mono$_{\Box }^i$) holds. Suppose that $(s_0, \dotsc , s_m) \le _{T} (s_0, \dotsc , s_m, s_{m+1}, \dotsc , s_k)$ and $(s_0, \dotsc , s_m, s_{m+1}, \dotsc , s_k) R_T (t_{0}, \dotsc , t_l)$ and $\bar{t}= (t_0, \dotsc , t_l, t_{l+1},\dotsc , t_p)$. By definition of $\le _T$ and $R_T$ we then have $s_m \le s_k$ and $s_{k } R t_l$. Since $t_l \le t_p$ and (Mono$_{\Box }^i$) holds in $\mathcal {K}$ we that $s_m R t_p$. By the definition of $R_T$ we get $(s_0, \dotsc , s_m) R_t (t_0, \dotsc , t_p)$.

The empty sequence is the root of $\mathcal {T}$. Clearly, any two $\le _T$-incomparable elements do not have an upper bound in $\le _T$. And since the elements of *T* are finite sequences any infinitely ascending chain does not have an upper bound. $\mathcal {T}$ is thus tree-like.

We define the embedding *f* by putting $f(s) = (s)$. To show that $\mathcal {K}, s \models \phi$ iff $\mathcal {T}, f(s) \models \phi$ we prove, by induction, the stronger claim that $\mathcal {K}, s \models \phi$ iff $\mathcal {T}, (s_0, \dotsc , s_{n-1}, s) \models \phi$ for all $s_0, s_1, \dotsc , s_{n_1}$ such that $s_0< s_1 \dotsc< s_{n-1} < s$.

The base case is immediate by the definition of ${\llbracket \phantom {x} \rrbracket }_{\mathcal {T}}$. The cases of $\wedge , \vee$ are immediate by the induction hypothesis.

Suppose that $\mathcal {K}, s \models \phi \rightarrow \psi$ and let $(s_0, \dotsc , s_{n-1}, s)$ be any sequence ending with *s*. Suppose that $\mathcal {T}, (s_0, \dotsc , s_{n-1}, s, t_0, \dotsc , t_m) \models \phi$. By the induction hypothesis $\mathcal {K}, t_m \models \phi$; by the definition of $\le _T$ we have $s \le t_m$, thus $\mathcal {K}, t_m \models \psi$. By the induction hypothesis $\mathcal {T}, (s_0, \dotsc , s_{n-1}, s, t_0, \dotsc , t_m) \models \psi$ and $\mathcal {T}, (s_0, \dotsc , s_{n-1}, s) \models \phi \rightarrow \psi$.

Suppose that $\mathcal {T}, (s_0, \dotsc , s_{n-1}, s) \models \phi \rightarrow \psi$ and suppose that $\mathcal {K}, t \models \phi$ for some $t \ge s$. Then by the induction hypothesis $\mathcal {T}, (s_0, \dotsc , s_{n-1}, s, t) \models \phi$ and so $\mathcal {T}, (s_0, \dotsc , s_{n-1}, s, t) \models \psi$. By the induction hypothesis $\mathcal {K}, t \models \psi$ and so $\mathcal {K}, s \models \phi \rightarrow \psi$.

Suppose $$\mathcal {K}, s \models \Box \phi $$ and let $$(s_0, \dotsc , s_{n-1}, s)$$ be any sequence ending with *s*. Suppose that $$(s_0, \dotsc , s_{n-1}, s) $$$$ R_T (t_0, \dotsc , t_m)$$. Then $$s R t_m$$ and so $$\mathcal {K}, t_m \models \phi $$. By the induction hypothesis we have $$\mathcal {T}, (t_0, \dotsc , t_m) \models \phi $$ and thus $$\mathcal {T}, (s_0, \dotsc , s_{n-1}, s) \models \Box \phi $$.

Suppose that $\mathcal {T}, (s_0, \dotsc , s_{n-1}, s) \models \Box \phi$. And suppose that *sRt*. Then $(s_0, \dotsc , s_{n-1}, s) R (t)$ and so $\mathcal {T}, (t) \models \phi$. By the induction hypothesis we then get $\mathcal {K}, t \models \phi$. And so $\mathcal {K}, s \models \Box \phi$.

It remains to be seen that this model satisfies the frame conditions in table 2 if $\mathcal {K}$ does. We do a few of the cases. If $\mathcal {K}$ is reflexive, then $() R_T ()$ was ensured by construction. The other cases are immediate.

To establish that $$\le _T$$ is weakly reflexive if < is weakly reflexive suppose that $$(s_0, \dotsc , s_{m}) \le _T (s_0, \dotsc , s_m, \dotsc , s_n)$$. Since $$\le $$ is weakly reflexive let $$s_{k}$$ and *t* be such that $$s_m R t$$ and $$s_{n} \le s_{k}$$ and $$t \le s_k$$. By (Mono$$_{\Box }^i$$) $$s_n R s_k$$. And thus $$(s_0, \dotsc , s_m) R_{T} (s_0,\dotsc , s_m, \dotsc , s_n, s_k)$$. But clearly $$(s_0, \dotsc ,$$$$ s_m, \dotsc , s_n) \le _{T} (s_0, \dotsc , s_m, \dotsc , s_n, s_k)$$. This shows that $$\le _T$$ is weakly reflexive.

For the case of **B** suppose that $(s_0, \dotsc , s_{m}) R_T (t_0, \dotsc , t_n)$. And let $(t_0, \dotsc , t_n) \le _T (t_{0}, \dotsc , t_n, \dotsc , t_k)$. We have $s_m R t_n$ and $t_n \le t_k$. Thus since $\le$ is symmetric$^{*}$ let $s_l \ge s_m$ be such that $t_k R s_l$. Then by definition of $R_T$ we have $(t_0, \dotsc , t_n, \dotsc , t_k) R_T (s_0, \dotsc , s_m, s_l)$ which is what we have to show.

For the case of $$\textbf{5}$$ suppose that *R* is Euclidean$$^{*}$$. Suppose that $$(s_0, \dotsc , s_m) R_T (t_0, \dotsc , t_n)$$. By definition $$s_m R t_n$$; since *R* is Euclidean$$^{*}$$ find $$s_k \ge s_{m}$$ such that for all *u* such that $$s_k R u$$ we have $$t_n R u$$. By definition of $$\le _T$$ and $$R_T$$ we have $$(s_0, \dotsc , s_m, s_k)$$ and for all *u* if $$(s_0, \dotsc , s_m, s_k) R_T (v_0, \dotsc ,$$ $$v_l, u)$$ then $$(t_0, \dotsc , t_n) R_T (v_0, \dotsc , v_l, u)$$. This shows that $$R_T$$ is Euclidean$$^{*}$$. $$\square $$

We next show to associate tree-like Kripke-models with exact modal models.

If $\langle S, \sqsubseteq \rangle$ is a partial order then $A \subseteq S$ is *downwards closed* if whenever $s \sqsubseteq t$ and $t \in A$, then $s \in A$. If *A* is downards closed then *A* is said to be *principal* if there is *t* such that $A= [t]= \left\{ s :s \sqsubseteq t\right\}$.

If $\mathcal {K}= \langle K, \le , R, {\llbracket \phantom {x} \rrbracket }_{\mathcal {K}}\rangle$ is a tree-like Kripke-model we define the associated exact model $\mathcal {E}/\mathcal {K}= \langle S, \sqsubseteq , C, N, {\llbracket \phantom {x} \rrbracket }_{\mathcal {E}}\rangle$ as follows.

- *S* is the set of downwards closed subsets of *T*;
- $\sqsubseteq$ is the subset relation on *S*;
- *C* is the set of non-principal downwards closed subsets.
- If $A=[s]$ is principal, then
  $$\begin{aligned} N([s])= &  \{P :\text { P is an upwards closed subset of } \\  &  S \text { such that for all } t \text { with } \\ &  s R t \text { there is } t_0 \le t \text { with } [t_{0}] \in P \} \end{aligned}$$
- If *A* is not principal, then $$N(A)=\{P :P \text { is an upwards} $$$$ \text {closed supset of } S \}$$
- We define ${\llbracket \phantom {x} \rrbracket }_{\mathcal {K}/\mathcal {E}}$ as follows.
  - $$[s] \in {\llbracket p \rrbracket }_{\mathcal {K}/\mathcal {E}}$$ iff $$s \in {\llbracket p \rrbracket }_{\mathcal {K}}$$;
  - $B \in {\llbracket p \rrbracket }_{\mathcal {K}/\mathcal {E}}$ for each non-principal *B*;
  - $${\llbracket \bot \rrbracket }_{\mathcal {K}/\mathcal {E}}= C$$.

#### Theorem A.14

(i) $\mathcal {E}/\mathcal {K}$ is an exact model.
(ii) If $\mathcal {K}$ satisfies a condition in table 2 then $\mathcal {E}/\mathcal {K}$ satisfies the corresponding condition in table 3.

#### Proof

For the first part, the proof that $\langle S, \sqsubseteq , C\rangle$ is a complete residuated partial order is as in Fine (2014). For definiteness, we give the proof here.

Let $\left\{ B_i:i \in I\right\}$ be a collection of downwards closed sets. If $\left\{ B_i :i \in I\right\}$ is empty, the root *r* of $\mathcal {K}$ will be the least upper bound. If $\left\{ B_i :i \in I\right\}$ is not empty $\bigcup _{i \in I} B_i$ is downwards closed and it is obviously the least upper bound.

We next establish that the residuation condition is met. Suppose *A*, *B* are downwards closed. Let $C= r \cup \left\{ [c] :c \in B {\setminus } A\right\}$. Clearly, *C* is downwards closed. We claim that $C \cup A \supseteq B$. Let $b \in B$. If $b \notin B {\setminus } A$, then $b \in A$ and thus $b \in A \cup C$. So suppose $b \in B {\setminus } A$, then $b \in C$. Thus $B \subseteq A \cup C$.

Suppose next that *D* is such that $D \cup A \supseteq B$. We claim that $C \subseteq D$. For let $c \in C$. Then there is $c_0$ such that $c \le c_0$ and $c_0 \in B {\setminus } A$. Since $D \cup A \supseteq B$ we have to have $c_0 \in D$, and thus $c \in D$ since *D* is downwards closed.

We now have to check that the conditions on *N* are satisfied.

(Nullity) and (Modal Closure) are immediate since *N* is defined on all members of *S*.

(Contradictory): if *A* is not principal, then $\left\{ S\right\} \in N(A)$, but $S \in C$.

(Ground Closure). If $A= [s]$ is principal, suppose $P_i, i \in I$ are in *N*([*s*]). Then for all *t* such that *sRt* there is $[t_{i}] \in P_{i}$, with $t_{i} \le t$ for all $i \in I$. But if $\left\{ P_i :i\in I\right\}$ grounds *Q* then $\bigcup _{i \in I} [t_{i}] \in Q$. But $\bigcup _{i \in I} [t_i] \subseteq [t]$. Thus $Q \in N([s])$. If *A* is non-principal, then every proposition is in *N*(*A*).

(Convexity): this is immediate since *N*(*A*) is in fact closed under containment.

(Monotonicity). Suppose $A \sqsubseteq B$. If $A=[s], B=[t]$ are both principal suppose $P \in N([s])$. Then for all *u* such that *sRu* there is $f(u) \le u$ such that $f(u) \in P$. Suppose *tRv*; by (Mono$_{\Box }^i$), *sRv*. Since $f(v) \le v$ it follows that $P \in N(t)$. Suppose next that $P \in N([t])$. We have $\left\{ r\right\} \in N([s])$ and thus by (Ground Closure) we have $P \cup \left\{ r\right\} \in N([s])$. But $\left\{ r\right\} \cup P \sqsubseteq P$. If *B* is non-principal the result is immediate.

(CN$^{+}$): this is immediate since *N*(*A*) is closed under containment.

We next turn to the second part of the theorem, taking the conditions in order.

**D**. Suppose that *R* is serial. Suppose that [*s*], [*t*] are comparable and that $P \in N([s])$. Since $\mathcal {K}$ is tree-like either $s \le t$ or $t \le s$. Without loss of generality, assume the former; we show that $\lnot P \notin N([t])$. By definition of *N* we have $P \in N([t])$. If $\lnot P$ was in *N*([*t*]) then, by (Ground Closure), $P \wedge \lnot P$ would be in *N*([*t*]). But since *R* is serial, let *u* be such that *tRu*. Then there is $u_0 \le u$ such that $u_0 \in P \wedge \lnot P$. But this is impossible, since *u* is consistent.

**T**. Suppose that *R* is reflexive and suppose that $P \in N([s])$. Then for all *t* such that *sRt*, there is $f(t)\le t$ with $[f(t)] \in P$. Since *R* is reflexive, *sRs* and thus $f(s) \le s$ with $[f(s)] \in P$. This is what we have to show.

**T**$_{\lnot \lnot }$. Suppose that $P \in N([s])$. Suppose [*s*] is compatible with a [*t*] such that $[t] \in \lnot P$. Since $\mathcal {K}$ is tree-like, without loss of generality, we may assume that $t \ge s$. By weak reflexivity find [*u*] such that $t \le u$ and $v \le u$ for some *v* such that *sRv*. But $[v] \in P$ and so $[u] \in P$. But since $[t] \in \lnot P$ also $[u] \in \lnot P$. But then $P \wedge \lnot P \in [u]$, but this is impossible since [*u*] is principal.

**B**: If $\emptyset \in N([s])$ we are done, so suppose $\emptyset \notin N([s])$. We show that [*s*] itself is an *s*-proponent. For suppose that $t \in M_{[s]}$. And suppose that $\lnot \left\{ s\right\} \in M_{[u]}$. Suppose $[t] \cup [u]$ is consistent. Without loss of generality, we may suppose that $t \le u$. Since *R* is symmetric$^{*}$ there is then $s_0 \ge s$ such that $u R s_0$. Since $\lnot \left\{ s\right\} \in M_u$ there is $c \in C$ such that $([s] \dashrightarrow c) \subseteq [s_0]$ but since $s \le s_0$ this means that $[s_0]$ is non-principal. Contradiction. We conclude that *t* is an *s*-guardian and thus that *s* itself is an *s*-proponent.

**B**$_{\lnot \lnot }$. The argument establishing $\textbf{B}_{\lnot \lnot }$ is similar.

The case of $\textbf{5}$ is immediate from the definition of *N*. $\square$

#### Proposition A.15

Let $\mathcal {T}=\langle T, \le , R, {\llbracket \phantom {x} \rrbracket }_{\mathcal {T}}\rangle$ be tree-like Kripke model, and $\mathcal {E}/\mathcal {T}$ its associated exact model. $\mathcal {K}/(\mathcal {E}/\mathcal {T})$ is isomorphic to $\mathcal {T}$.

#### Proof

Define $$f :\mathcal {T} \rightarrow \mathcal {K}/(\mathcal {E}/\mathcal {T})$$ by $$f(s)= [s]$$.

Clearly, *f* is a bijection; we also have $s \le t$ iff $[s] \subseteq [t]$; and we have $s \in {\llbracket p \rrbracket }_{\mathcal {T}}$ iff $[s] \in {\llbracket p \rrbracket }_{\mathcal {K}/(\mathcal {E}/\mathcal {T})}$.

It remains to be shown that *sRt* iff $[s] R_{\mathcal {K}/({\mathcal {E}/\mathcal {T}})} [t]$. We clearly have that if *sRt* then $[t] \in M_{\mathcal {E}/\mathcal {T}}([s])$. For the other direction, suppose that $[t] \in M_{\mathcal {E}/\mathcal {T}}([s])$. There is $t_0$ such that $s R t_0$ and $t_{0} \le t$. But since [*t*] is principal, $t \in T$ and so *sRt* by (Mono$_{\Box }^i$).

By definition of $$R_{\mathcal {K}/(\mathcal {E}/\mathcal {T})}$$ we also have $$ M_{\mathcal {E}/\mathcal {T}}([s]) \text { iff } [s] $$$$ R_{\mathcal {K}/(\mathcal {E}/\mathcal {T})} [t]$$. This establishes the result. $$\square $$

We can now prove completeness result.

#### Proof of Theorem A.11

Suppose that $\phi$ does not follow from $\Gamma$ in **IntK**$S_1 \dotsc S_n$. Let $\mathcal {T}$ be a tree-like Kripke model satisfying conditions $S_1, \dotsc , S_n$ and let *s* be a state in $\mathcal {T}$ such that $\mathcal {T}, s \models \Gamma$ but $\mathcal {T}, s \not \models \phi$.

Let $\mathcal {E}/\mathcal {T}$ be the associated exact model and let $\mathcal {K}/(\mathcal {E}/\mathcal {T})$ be the inexact companion. We then have

$$\begin{aligned} \mathcal {T}, s \models \phi \text { iff } \mathcal {K}/(\mathcal {E}/\mathcal {T}), [s] \models \phi \text { iff } \mathcal {E}/\mathcal {T}, [s] \mathrel {\Vdash _{\text {i}}}\phi \end{aligned}$$

But then $\mathcal {E}/\mathcal {T}, [s] \mathrel {\Vdash _{\text {i}}}\Gamma$ but , showing that $\Gamma \nVdash ^{S_1, \dotsc , S_n}_{i12} \phi$. $\square$

### A.9 World-validity

We formulate classical S5 as follows. The axioms and rules of **IntK**$_{\Box }$; the axioms **T**, **4**, **5**; all instances of $\overbrace{\Box \dotsc \Box }^{n} (\phi \vee \lnot \phi )$.

#### Theorem A.16

All theorems of classical S5 are inexactly verified at each world in a modally classical model satisfying the $\textbf{T}_{\lnot , \lnot }, {\textbf {5}}_{\lnot \lnot }, {\textbf {4}}$ conditions.

#### Proof

Clearly all instances of $\Box P \rightarrow \lnot \lnot P$, $\Box P \rightarrow \lnot \lnot \Box P$ and $\lnot \Box P \rightarrow \lnot \lnot \Box \lnot \Box P$ are verified by the null state in a model satisfying the ${\textbf {T}}_{\lnot \lnot ,}, {\textbf {5}}_{\lnot \lnot }$ and $\textbf{4}$-conditions. Thus all the instances of **T**,**4**, **5** are inexactly verified at every world.

It thus suffices to show that all instances of $\overbrace{\Box \dotsc \Box (\phi \vee \lnot \phi )}^n$ are inexactly verified at each world.

Observe first that excluded middle holds at each world. For let *w* be a world and suppose that *w* does not contain a verifier for *P*. Then *w* contains a conditional connection $s \dashrightarrow c$, $c \in C$, for each verifier *s* of *P* and so *w* inexactly verifies $\lnot P$.

So let *w* be a world. Since the state space is modally classical, $M_w$ contains only worlds. Thus $w \mathrel {\Vdash _{\text {i}}}\Box (\phi \vee \lnot \phi )$ for each *w*. By induction, $w \mathrel {\Vdash _{\text {i}}}\overbrace{\Box \dotsc \Box }^n (\phi \vee \lnot \phi )$. $\square$

## Footnotes

1 A parallel question arises for possibilized propositions. For reasons of simplicity, we deal only with the truthmakers for necessitated propositions. However, given the intuitionistic truthmaker theory adopted here, in a fuller treatment one should not treat necessity and possibility as duals.

2 Some answers include: (facts about) essences, dispositions, recombination, possible worlds, conventions, and laws of metaphysics.

3 The paper is in the tradition of work in exact truthmaker semantics pioneered in Fine 2012c; 2014; 2017a; b; 2021; 2012a; c. Previous attempts at truthmaker semantics for modal logic are Hale Hale 2020, ch. 7, Zylstra 2019, and Rosella 2019. The work on this paper was complete before the publication of Kim forthcoming. A comparison between the two approaches has to await another occasion.

4 We will use “verifier” and “truthmaker” interchangeably; similarly, for “makes true” and “verifies”.

5 Sometimes one also requires that a fusion of a truthmaker for *P* with a truthmaker for *Q* is a truthmaker for $P \vee Q$. But the issues raised by this “amalgamating” clause will not matter for the purposes of this paper.

6 See also Linnebo 2022 for more on the idea that (semi)-intuitionistic logic has a special metaphysical status.

7 Some might argue that one should express the insubstantiality of the truths of intuitionistic logic in terms of *ground* rather than truthmaking (Fine 2012b, pp. 43–46). The insubstantiality claim would then be that the truths of intuitionistic logic are *zero-grounded*. See Donaldson 2020 for an introduction to this idea.

8 I am very grateful to two anonymous referees for raising the objections that lead to this section.

9 For a different clause see Fine 2020, p 160. We here have to face up to a the fact that in a bilateral framework there no “canonical” way of deriving the falsemaking clause from the truthmaking clause (cf. Fine 2017a, pp. 633–634).

10 The bilateral account of negation is not the only other treatment of negation in the literature on truthmaking. Fine (2017a) develops an account of negation using an *exclusion relation* and Plebani et al. (2022) develop a related account using *incompatibility* relations. There are ways of developing these accounts where the conditional $\lnot \lnot P \rightarrow P$ ends up being null-verified. But given that both these accounts retain the standard truthmaking clauses for disjunction, some instances of excluded middle will not be null-verified.

11 For an introduction to neighborhood semantics in a possible worlds setting, see e.g., Pacuit (2017).

12 The phenomenon of non-modal states means that we should not take propositions to be *convex* in the sense that if $s, t, \in P$ and $s \sqsubseteq u \sqsubseteq t$, then $u \in P$. Consider the proposition that $0=0$ or the pebble is on the beach. Williamson can know this proposition in virtue of knowing that $0=0$; call the state that realizes this *s*. He can also know it in virtue of knowing that the pebble is on the beach; call the state that realizes this *t*. Finally, he can know it in virtue of knowing both that $0=0$ and that the pebble is on the beach. A state that realizes this is $s \sqcup t$—the fusion of *s* and *t*. *t* contains the state $t_0$ of the pebble’s being on the beach. Convexity requires that $s \sqcup t_0$ is a state that makes Williamson know that $0=0$ or the pebble is on the beach. But this seem incorrect.

13 For remarks on the distinction between modal and non-modal states see further Fine’s introduction to Hale 2020, pp. 6–8.

14 Note that we allow the case where $P_{0}=P_1=P_2 = \dotsc$.

15 For some interpretations of $\Box$ (Nullity) is questionable. For instance, we might not want (Nullity) for the modality “it is known by Williamson that $\dotsc$” since this would treat Williamson as logically omniscient.

16 Suppose one adopts a bilateral treatment of negation. It then seems reasonable to expect that $\Box \top$ as well as all instances of (**K**$_{\Box }$) are null-valid. It would be of considerable interest to determine exactly which modal principles will be null-valid in such a bilateral system.

17 Hale (2020, pp. 128–129) has, working in a bilateral framework, sketched a different relational approach. Various problems with Hale’s account are pointed out by Fine in this introduction to Hale 2020, pp. 6–8. A couple of further problems should be mentioned here. Hale’s initial account is that a state *s* is a truthmaker for $\Box P$ iff no state accessible from *s* is a falsemaker for *P*. This runs into the following problem. Suppose *s* is a truthmaker for $\Box (P \wedge Q)$. Then no state accessible from *s* is a falsemaker for $P \wedge Q$. So no state accessible from *s* is an exact falsemaker for either *P* or *Q*. But then *s* is a truthmaker for both $\Box P$ and $\Box Q$. But it is not plausible that *every* truthmaker for the necessity of a conjunction is a truthmaker for the necessity of the conjuncts. For reasons like this Hale considers adding the requirement that some state accessible from *s* is an exact truthmaker for *P*. While this avoids the above problem, one now has to impose a requirement like the problematic (CN) discussed below to ensure that ($\Box _{\text {Down}}$) holds. A further problem with Hale’s account can be brought out as follows. Hale (2020, P. 129) asserts that if one takes $\Box$ “to express absolute necessity [...] conforming to S5 laws, then reference to [the accessibility relation] may be suppressed” and one can take *s* to make $\Box P$ true iff no possible state falsifies *P*. Suppose now that every possible state is contained in a world—that is, a maximal possible state. Now let *W* be the proposition that is made exactly true by each world. Then every modal state is a truthmaker for $\Box W$. But even if one accepts classical logic and holds that every state is contained in a world, one might not want *every* modal state to be a truthmaker for $\Box W$.

18 Many thanks to an anonymous referee for forcing me to clarify the relationship between (CN) and ($\Box _{\text {Down}}$).

19 The law, in all its 274 pages, deals with more than that: it governs subsidies for clean energy and electric cars, maximum prices for insulin and others drugs, clean-up of superfund sites, corporate tax reform, $\dotsc$. The full text of the law is available at https://www.congress.gov/117/plaws/publ169/PLAW-117publ169.pdf. Thanks to for suggesting this example.

20 The proposition *B* is not the same as the proposition that it is a law that the price cap is $35. These propositions are not even intensionally equivalent. Even if it is a law that the price cap is $35 your pharmacist might make a mistake and charge you more; and even when the law does not cap the price the pharmaceutical company could be in a generous mood and voluntarily cap the price. (Similar points apply to *A* and the proposition that it is a law that the tax credit is $7500.)

21 It is, again, important to distinguish the propositions *A*, *B* from the propositions that they are laws. The very existence of the ira shows that a way for it to be a law that *A* is a way for it to be a law that *B* (as well as a way for it to be a law that $A \wedge B$.)

22 For a version of this view see Linnebo (2008) and with more metaphysical elaboration Litland (2022).

23 cf. Linnebo 2008, pp. 78–79 on how the natural numbers *weakly depend* on each other.

24 This problem also arises for $\textbf{5}$. There does not seem to be a reason for taking a state that verifies $\lnot \Box p$ itself to be a modal state. Interestingly, the situation would be different if one had a primitive possibility operator $\Diamond$. The **5**-principle would then be $\Diamond P \rightarrow \Box \Diamond P$, and of course a state that verifies $\Diamond P$ would be a modal state.

25 Here it is important not to confuse the proposition which is necessary with the proposition that it is necessary. Of course, the proposition that it is raining or not raining is made true by the state of its raining. What is that issue is whether the proposition that it is *necessary* that it is raining or not raining is made true by this.

26 There is a relation here to the distinction between unworldly (or transcendental) facts and worldly (or mundane) facts drawn in Fine (2005). It would be interesting to explore if one can use truthmaker semantics to model the distinction between Fine’s grades of necessity (Fine 2005, pp. 326–328).

27 The same phenomenon is illustrated by nomological necessity. It might be a law of nature that *P*. For *humeans* it is partly because it is the case that *P* that it is a law of nature that *P*. But for non-humeans this is not so: its being the case that *P* is not part of what it is for it to be a law that *P*. Non-humeans thus should reject the null-validity of $\textbf{T}$. For related ideas, see Shumener (2022).

28 We set aside the worries about **4** due to Chandler (1976) and Salmon (1989).

29 If one had a separate possibility operator this would also ensure that $\Diamond P \rightarrow \Box \Diamond P$ is $0$-valid.

30 This characterization of what ensures world-validity assumes the intuitionist’s treatment of negation. It is an interesting question how this should be done if one adopts a bilateral treatment of negation.

31 For more on this idea see Elgin (2021). Related ideas have been explored a lot recently, though not in the context of truthmaker semantics. See, e.g., Hawke et al. (2020) Hawke and Berto (2021).

32 It should be mentioned that Zylstra (2019) already has proposed a truthmaker semantics for essence. Unfortunately, as shown by Vogt (2021), his account is unsatisfactory.
